# Whole Exome/Genome Sequencing Joint Analysis of a Family with Oligogenic Familial Hypercholesterolemia

**DOI:** 10.3390/metabo12030262

**Published:** 2022-03-18

**Authors:** Youmna Ghaleb, Sandy Elbitar, Anne Philippi, Petra El Khoury, Yara Azar, Miangaly Andrianirina, Alexia Loste, Yara Abou-Khalil, Gaël Nicolas, Marie Le Borgne, Philippe Moulin, Mathilde Di-Filippo, Sybil Charrière, Michel Farnier, Cécile Yelnick, Valérie Carreau, Jean Ferrières, Jean-Michel Lecerf, Alexa Derksen, Geneviève Bernard, Marie-Soleil Gauthier, Benoit Coulombe, Dieter Lütjohann, Bertrand Fin, Anne Boland, Robert Olaso, Jean-François Deleuze, Jean-Pierre Rabès, Catherine Boileau, Marianne Abifadel, Mathilde Varret

**Affiliations:** 1 INSERM, Laboratory for Vascular Translational Science (LVTS), F-75018 Paris, France; youmna.ghaleb@inserm.fr (Y.G.); sandy.el-bitar@inserm.fr (S.E.); petra.el-khoury@inserm.fr (P.E.K.); yara.azar@inserm.fr (Y.A.); miangaly.andrianirina@inserm.fr (M.A.); alexia.loste@inserm.fr (A.L.); yara.abou-khalil@inserm.fr (Y.A.-K.); marie.le-borgne-moynier@inserm.fr (M.L.B.); jean-pierre.rabes@aphp.fr (J.-P.R.); catherine.boileau@inserm.fr (C.B.); marianne.abi-fadel@inserm.fr (M.A.); 2 Laboratory of Biochemistry and Molecular Therapeutics (LBTM), Faculty of Pharmacy, Pôle Technologie-Santé (PTS), Saint-Joseph University, Beirut 1004 2020, Lebanon; 3 Institut Cochin, Bâtiment Faculté Inserm U1016, Cnrs UMR8104, Université de Paris Faculté de Médecine, F-75014 Paris, France; anne.philippi@inserm.fr; 4 Laboratory for Vascular Translational Science, Paris Cité University, Sorbonne Paris Nord University, F-75013 Paris, France; gael.nicolas@inserm.fr; 5 INSERM U1149, CNRS ERL 8252, Centre de Recherche sur l’Inflammation, F-75018 Paris, France; 6 Department of Endocrinology, Nutrition and Metabolic Diseases, Hospices Civils de Lyon, Louis Pradel Cardiovascular Hospital, F-69500 Bron, France; philippe.moulin@chu-lyon.fr (P.M.); sybil.charriere@chu-lyon.fr (S.C.); 7 CarMen Laboratory, INSERM U1060, INRAE U1397, Université Lyon 1, F-69921 Oullins, France; mathilde.di-filippo@chu-lyon.fr; 8 Hospices Civils de Lyon, Department of Biochemistry and Molecular Biology, F-69500 Bron, France; 9 EA 7460 Physiopathologie et Epidémiologie Cérébro-Cardiovasculaires (PEC2), Université de Bourgogne-Franche Comté, F-21078 Dijon, France; farnier.michel@orange.fr; 10 Département de Médecine Interne et Immunologie Clinique Centre de Référence des Maladies Auto-Immunes Systémiques Rares du Nord et Nord-Ouest de France (CeRAINO) CHU de Lille, F-59037 Lille, France; cecile.yelnik@chru-lille.fr; 11 U1167 Risk Factors and Molecular Determinants of Aging-Related Diseases, Inserm CHU de Lille, Lille University, F-59000 Lille, France; 12 Department of Endocrinology and Prevention of Cardiovascular Disease, Institute of Cardio Metabolism and Nutrition (ICAN), La Pitié-Salpêtrière Hospital, AP-HP, F-75005 Paris, France; valerie.carreau@aphp.fr; 13 Department of Cardiology, Toulouse Rangueil University Hospital, UMR 1295 INSERM, F-31400 Toulouse, France; jean.ferrieres@univ-tlse3.fr; 14 Nutrition Department, Institut Pasteur de Lille, CEDEX, F-59019 Lille, France; jean-michel.lecerf@pasteur-lille.fr; 15 Child Health and Human Development Program, Research Institute of the McGill University Health Centre, Montréal, QC H3A 0G4, Canada; alexa.derksen@mail.mcgill.ca (A.D.); genevieve.bernard@mcgill.ca (G.B.); 16 Translational Proteomics Laboratory, Institut de Recherches Cliniques de Montréal, Montréal, QC H2W 1R7, Canada; marie-soleil.gauthier@ircm.qc.ca (M.-S.G.); benoit.coulombe@ircm.qc.ca (B.C.); 17 Department of Neurology and Neurosurgery, McGill University, Montréal, QC H3A 0G4, Canada; 18 Department of Pediatrics, McGill University, Montréal, QC H3A 0G4, Canada; 19 Department of Human Genetics, McGill University, Montréal, QC H3A 0G4, Canada; 20 Division of Medical Genetics, Department of Specialized Medicine, McGill University Health Centre, Montréal, QC H4A 3J1, Canada; 21 Department of Biochemistry and Molecular Medicine, Université de Montréal, Montréal, QC H3T 1J4, Canada; 22 Institute of Clinical Chemistry and Clinical Pharmacology, University Hospital Bonn, F-53127 Bonn, Germany; dieter.luetjohann@ukbonn.de; 23 CEA, Centre National de Recherche en Génomique Humaine, Laboratory of Excellence GENMED (Medical Genomics), Paris-Saclay University, F-91057 Evry, France; bertrand.fin@cng.fr (B.F.); boland@cng.fr (A.B.); robert.olaso@cng.fr (R.O.); deleuze@cng.fr (J.-F.D.); 24 Centre d’Etude du Polymorphisme Humain, Fondation Jean Dausset, F-75019 Paris, France; 25 Department of Biochemistry and Molecular Genetics, Ambroise Paré University Hospital (APHP), Université Paris-Saclay, F-92104 Boulogne-Billancourt, France; 26 UFR (Unite de Formation et de Recherche) Simone Veil-Santé, Versailles-Saint-Quentin-en-Yvelines University, F-78180 Montigny-le-Bretonneux, France; 27 Genetic Department, AP-HP, Hôpital Bichat, F-75018 Paris, France

**Keywords:** autosomal dominant hypercholesterolemia, linkage analysis, next-generation sequencing, LDL uptake, *CYP7A1*, *LRP6*, *LDLRAP1*, protein structural models, polygenic risk score, oligogenic hypercholesterolemia

## Abstract

Autosomal Dominant Hypercholesterolemia (ADH) is a genetic disorder caused by pathogenic variants in *LDLR*, *APOB*, *PCSK9* and *APOE* genes. We sought to identify new candidate genes responsible for the ADH phenotype in patients without pathogenic variants in the known ADH-causing genes by focusing on a French family with affected and non-affected members who presented a high ADH polygenic risk score (wPRS). Linkage analysis, whole exome and whole genome sequencing resulted in the identification of variants p.(Pro398Ala) in *CYP7A1*, p.(Val1382Phe) in *LRP6* and p.(Ser202His) in *LDLRAP1*. A total of 6 other variants were identified in 6 of 160 unrelated ADH probands: p.(Ala13Val) and p.(Aps347Asn) in *CYP7A1*; p.(Tyr972Cys), p.(Thr1479Ile) and p.(Ser1612Phe) in *LRP6*; and p.(Ser202LeufsTer19) in *LDLRAP1*. All six probands presented a moderate wPRS. Serum analyses of carriers of the p.(Pro398Ala) variant in *CYP7A1* showed no differences in the synthesis of bile acids compared to the serums of non-carriers. Functional studies of the four *LRP6* mutants in HEK293T cells resulted in contradictory results excluding a major effect of each variant alone. Within the family, none of the heterozygous for only the *LDLRAP1* p.(Ser202His) variant presented ADH. Altogether, each variant individually does not result in elevated LDL-C; however, the oligogenic combination of two or three variants reveals the ADH phenotype.

## 1. Introduction

Autosomal Dominant Hypercholesterolemia (ADH) is a genetic disorder affecting lipoprotein metabolism that is characterized by the high plasma levels of low-density lipoprotein (LDL) due to its reduced catabolism [1]. Lifelong exposure of arteries to elevated levels of cholesterol promotes early atherosclerotic plaque development and premature cardiovascular disease (CVD) increasing the risk of heart attack, stroke and peripheral vascular disease [2]. In addition to CVD, individuals with ADH may have extravascular deposits, such as tendinous xanthomas, xanthelasma or corneal arcus [3]. 

ADH is one of the most frequent genetic diseases with a prevalence of 1 in 313 in the general population [4] and more frequent in populations with founder effects (i.e., French Canadians, Afrikaners, and Lebanese) [5]. ADH is caused by mutations in the low-density lipoprotein receptor gene (*LDLR*) at 19p13.3 (OMIM #143890, #606945) [6,7], the apolipoprotein B gene (*APOB*) at 2p23–p24 (OMIM #107730, #144010) [8], the proprotein convertase subtilisin/kexin type 9 gene (*PCSK9*) at 1p32.3 (OMIM # 607786) [9] and the apolipoprotein E gene (*APOE*) at 19q13.32 (OMIM #107741) [10]. The low-density lipoprotein receptor adaptor protein 1 gene (*LDLRAP1*) at 1p36.11 (OMIM # 605747) is the only gene responsible for the recessive form of hypercholesterolemia identified to date [11]. 

Mutations in the *LDLR* gene are the most frequent cause of ADH (80–85% of the cases), and more than 3000 variants have been reported [3]. The LDL receptor removes LDL particles from plasma, and alterations in its gene (*LDLR*) are commonly associated with high LDL-C levels [3]. Mutations in *APOB* are the second most frequent cause of ADH (5 to 10% of the cases). Apolipoprotein B is the ligand of LDL receptor and ADH-causative variants in *APOB* prevent or alter LDL binding to the LDL receptor [12]. Less frequent (2% of the cases) are *PCSK9* gain-of-function mutations that are associated with high LDL-C levels due to increased lysosomal LDL receptor degradation [9,13]. The least frequent are mutations in *APOE* (1% of the cases) that also affect LDL binding to the LDL receptor [14].

In addition to the four major genes, ADH-causative defects are identified in “minor genes” [5]. While involved in recessive forms of hypercholesterolemia, heterozygous variants in *LDLRAP1* have been shown to increase LDL-C levels [15]. Putative pathogenic variants in the adenosine triphosphate-binding cassette transporter G5/G8 genes (*ABCG5*/*ABCG8*) that are involved in sitosterolemia (OMIM #210250), were found in 2.4% of subjects in an ADH Dutch cohort [16]. Possible pathogenic variants in the lysosomal acid lipase A gene (*LIPA*) that are involved in recessive lysosomal acid lipase deficiency, LAL (OMIM #278000), were found in 2.2% of subjects in an ADH Portuguese cohort [17]. In addition, a frameshift variant in the gene encoding the cholesterol 7α-hydroxylase (CYP7A1) segregates with high LDL-C levels in a large family [18]. CYP7A1 is an enzyme that catalyzes the rate-limiting step in the conversion of cholesterol to bile acids in the liver (OMIM #118455). Several rare missense variants in the gene encoding LDL receptor-related protein 6 (*LRP6*), which plays a crucial role in lipoprotein endocytosis and is an essential co-receptor in the Wnt/ß-catenin signaling pathway (OMIM #603507) [19,20], are linked to metabolic syndrome, high LDL-C levels, and early onset of CVD [21,22,23]. Rare variants in the gene encoding the patatin-like phospholipase domain-containing 5 (*PNPLA5*) were significantly associated with higher LDL-C levels in an American cohort [24].

Mutations identified in genes causing ADH account for approximately 80% of cases [25]. Based on weighted polygenic risk score (wPRS) calculations [26], a polygenic origin may account for 36% of nonmutated hypercholesterolemic patients [27]. This indicates the existence of a greater level of genetic heterogeneity in ADH and the involvement of unknown genes [28]. Between monogenic and polygenic forms of ADH, digenic forms were reported with double-heterozygous carriers of mutations in two ADH major genes (*LDLR*/*APOB*, *LDLR*/*PCK9* [29] or *APOB*/*PCSK9* [30]), or in one major gene (*LDLR*, *APOB*, *PCSK9*, *APOE*) and one minor gene (*ABCG5*, *ABCG8*, *LDLRAP1*) [31]. To our knowledge, oligogenic forms with three variants in non-conventional ADH genes have not been reported.

We conducted this study in 1 French ADH family and in 160 unrelated hypercholesterolemic French probands in whom no pathogenic variant was found in the four ADH major genes. Our aim is to identify a new gene responsible for the ADH phenotype, which would allow a better understanding of ADH underlying causes and pathophysiology and would reveal new therapeutic targets.

## 2. Results

### 2.1. Patients Characteristics

**Figure 1:**
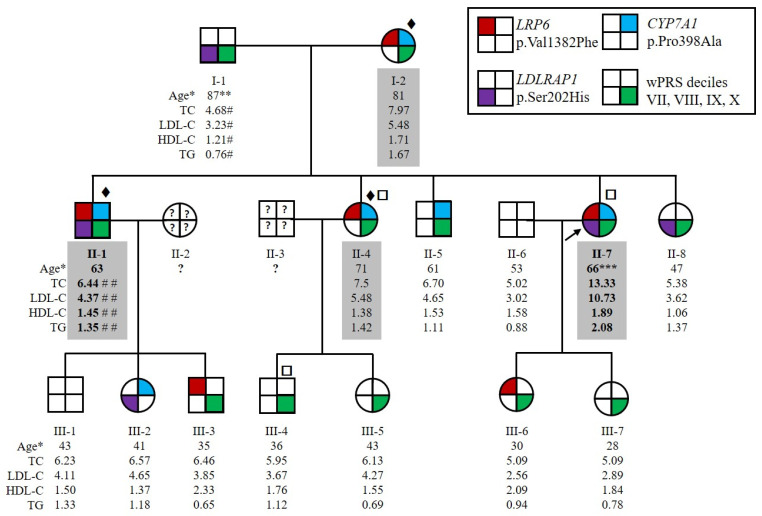
**Pedigree of the family HC438 with the segregation of p.(Val1382Phe) variant in *LRP6*, p.(Pro398Ala) variant in *CYP7A1* and p.(Ser202His) variant in *LDLRAP1* and the weighted Polygenic Risk Score (wPRS).** The proband II-7 is indicated by the black arrow. Squares and circles represent men and women, respectively. Affected family members are indicated by values highlighted in gray. More severely affected family members are indicated by bold values. * Age at lipid measurement in years. ** Myocardial infarction at 75 years old. *** Severe atheroma. # Under ciprofibrate. ## Under 5 mg rosuvastatin treatment. Patients for whom ♦ whole exome, □ whole genome sequencing was performed. Lipid values in mmol/L: TC for total cholesterol; LDL-C for LDL cholesterol; HDL-C for HDL cholesterol; TGs for triglycerides.

The family HC438 recruited for this study contained four affected members over two generations, which was large enough to conduct statistically meaningful genetic studies to identify new genes causing ADH. The index case for this family, II-7 (Figure 1), had a total cholesterol level of 9.33 mmol/L and LDL-C of 6.67 mmol/L before lipid-lowering therapy at the age of 56 years (both above the 90th percentile in agreement with inclusion criteria for affected ADH subjects, see Section 4.1). Her levels reached 13.33 mmol/L for total cholesterol and 10.73 mmol/L for LDL-C without treatment at 66 years of age, suggesting a homozygous form of ADH. She suffered from severe atheroma and had no extravascular cholesterol deposits. She also had a family history of hypercholesterolemia and cardiovascular heart disease. All willing members of the family were recruited, resulting in an expansion to 15 individuals over 3 generations, including 4 affected (I-2, II-1, II-4 and II-7) and 11 normocholesterolemic (i.e., normal cholesterol) individuals (I-1, II-5, II-6, II-8, all 3rd generation) (Figure 1). Both parents of the index case II-7 had elevated LDL-C or CVD. The father, I-1, had a myocardial infarction at the age of 75 years and an LDL-C level of 3.23 mmol/L under ciprofibrate treatment at the age of 87 years. We therefore considered him to be unaffected. The mother, I-2, had an LDL-C level of 5.48 mmol/L under diet at the age of 81 years. The LDL-C level of subject II-1 at the age of 63 years was 4.37 mmol/L under 5 mg rosuvastatin treatment and was estimated to be 7.95 mmol/L without treatment [32]. Patients II-1 and II-7 had higher LDL-C levels than their sister II-4. Therefore, because of the absence of consanguinity of their parents, patients II-1 and II-7 probably inherited two traits, one from each parent, whereas II-4 inherited the disease from her affected mother only (Figure 1). None of the affected family members presented extravascular cholesterol deposits or CVD, except subjects I-1 and II-7 who had CVD. 

In addition to the HC438 family, a cohort of 160 non-LDLR/non-APOB/non-PCSK9/non-APOE French ADH probands was collected. Lipid values before treatment were available for 98 probands (62.24% women, age: 52.5 ± 19 years): 7.96 ± 1.76 mmol/L for total cholesterol, 5.9 ± 1.24 mmol/L for LDL-C, 1.58 ± 0.71 mmol/L for HDL-C and 1.57 ± 1.1 mmol/L for triglycerides. The wPRS was calculated and 55.2% of the cohort probands were in the top four deciles (VII, VIII, IX and X) of the WHII reference cohort (Figure S1).

### 2.2. Three Candidate Variants Were Identified by Whole Genome Sequencing, Whole Exome Sequencing and Positional Cloning

We conducted whole genome sequencing (WGS) and whole exome sequencing (WES) of family HC438 and data were analyzed conjointly. Using a dedicated in-house Python pipeline, 22 variants within the family meeting the filtering criteria (variant quality, frequency, localization, segregation with ADH, predicted consequence and genotype quality; see Section 4.2) were identified in the heterozygous state. Four variants were transmitted from the mother (I-2) to the three affected children (II-1, II-4 and II-7) corresponding to three missense variants in *CYP7A1*, *KIFC2* and *LRP6*, and one intronic variant in *SLC39A4*. Three missense variants in the *LDLRAP1*, *GOLGA4* and *AP2A1* genes were transmitted from the father (I-1) to the two more severely affected children (II-1 and II-7). Three variants were transmitted from the mother (I-2) to the two more severely affected children (II-1 and II-7) corresponding to one missense variant in *MOGAT2* and two intronic variants in *PEX19* and *TSC2*. Finally, 12 UTR variants were transmitted by either parent to the 3 affected children (II-1, II-4 and II-7) (Table S1).

We performed positional cloning using a genome-wide scan of the entire family with 1072 polymorphic microsatellite markers. Parametric linkage analyses were performed under four hypotheses: (1) a paternal trait inherited by the three affected children, (2) a paternal trait inherited by the two more severely affected children, (3) a maternal trait inherited by the three affected children, and (4) a maternal trait inherited by the two more severely affected children (Table S2). The linkage analysis showed a maximum expected logarithm of odds score (ELOD) between 1.61 and 2.00 for the HC438 family, which only allowed significant exclusion of the rs62371472 variant in the *AP3S1* gene and the probable exclusion of the rs541351955 variant in the *SMAP2* gene from paternal and maternal inheritance. The variants *CD59* 3′UTR, *LDLRAP1*-p.(Ser202His) and *GOLGA4*-p.(Arg1494Ile) are probably linked to the disease with a paternal inheritance. The variants *CYP7A1*-p.(Pro398Ala), *LRP6*-p.(Val1382Phe) and *SLC2A3* 3′UTR, as well as *TSC2* intronic variants, are probably linked to the disease with a maternal inheritance. These seven genes with nonsynonymous variants are probably linked to paternally or maternally inherited traits.

Among the seven genes, we selected the three variants in the ADH minor genes *CYP7A1*, *LRP6* and *LDLRAP1* for further analysis. We performed segregation analysis by Sanger sequencing of all recruited family members. The variants p.(Val1382Phe) in *LRP6* and p.(Pro398Ala) in *CYP7A1* are transmitted from the mother (I-2) to the three affected children (II-1, II-4 and II-7). The variants are also transmitted to the two unaffected members (III-3 and III-6 for *LRP6* variant, II-8 and III-2 for *CYP7A1* variant), indicating incomplete penetrance of the associated phenotype (Figure 1). The p.(Ser202His) variant in *LDLRAP1* was transmitted from the father (I-1) to the two more severely affected children (II-1, II-7); however, it is also transmitted to two unaffected members (II-8 and III-2) (Figure 1). Thus, the two more severely affected family members (II-1 and II-7) carried the three variants. In silico analysis of these variants is detailed in Table 1.

### 2.3. Identification of New Variants in CYP7A1, LDLRAP1 and LRP6

**Figure 2:**
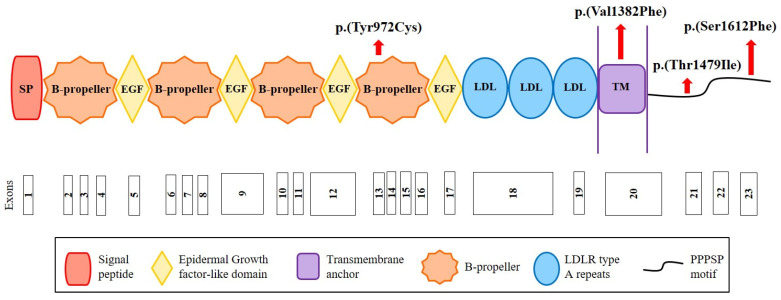
**Structure of the LRP6 receptor and position of the identified variants**. The LRP6 receptor contains the following structural motifs: signal peptide (SP), 4 β-propeller domains, 4 EGF-like domains (involved in the pH-dependent release of ligands in endosome), 3 LDLR type A repeats (responsible for the binding of ligands), a transmembrane anchor (binds the receptor to the cell membrane), and a cytoplasmic domain with PPPSP motifs (2 motifs at position 1487 and 1604 that allow the receptor to function in the Wnt/β-catenin pathway). Red arrows indicate the position of the variants identified in this study. Figure built from data from UniProt (www.uniprot.org (accessed on 12 October 2020)) and Ensembl (www.ensembl.org/index.html (accessed on 12 October 2020)) databases.

We sequenced the *CYP7A1*, *LDLRAP1* and *LRP6* genes in the cohort of 160 French hypercholesterolemic probands in whom mutations in *LDLR*, *APOB*, *PCSK9* and *APOE* were excluded. We replicated the *CYP7A1* finding by identifying the same p.(Pro398Ala) mutation in a 71-year-old man who presented total cholesterol of 7.52 mmol/L and LDL-C of 5.69 mmol/L. Among other cohort members, we identified the two variants p.(Ala13Val) and p.(Asp347Asn) in this gene and the four rare missense variants p.(Tyr972Cys), p.(Thr1479Ile) and p.(Ser1612Phe) in *LRP6* and p.(Ser202LeufsTer19) in *LDLRAP1* (Table 1 and Table 2). The p.(Tyr972Cys) variant in *LRP6* is located in the fourth ß-propeller domain of the LRP6 receptor, whereas two other variants are in the intracellular domain (Figure 2). The substituted residues are highly conserved among species from human to zebrafish (Figure S1). The p.(Val1382Phe) variant in *LRP6* is located in the transmembrane domain of LRP6 (Figure 2) in a conserved region (Figure S2).

**Figure 3:**
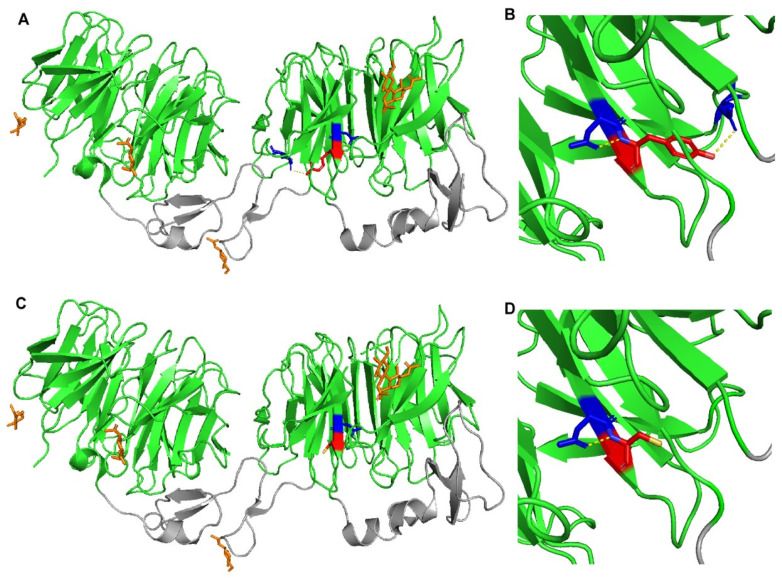
**Crystal structure of wild-type and mutant LRP6-E3E4 with β-propeller domains (green) and epidermal growth factor (EGF)-like domains (gray)**. (**A**,**B**) LRP6-E3E4 Tyr972 residue (red) has polar contacts (yellow dotted lines) with Asp971 and Glu993 (blue). (**C**,**D**) LRP6-E3E4 mutant Cys972 residue (red) has a polar contact (yellow dotted line) only with Asp971 (blue).

The use of PyMOL to model the effects of the p.(Tyr972Cys) in LRP6-E3E4 (PMB ID 3S8Z) showed that this missense variant results in the loss of a polar interaction with a neighboring glutamic acid residue at position 993 (Figure 3). As a result, this mutant could result in altered stability of LRP6 protein and/or its ability to uptake LDL. A crystal structure of LRP6 containing its transmembrane and intracellular domains is not available, so the variants p.(Val1382Phe), p.(Thr1479Ile), p.(Tyr1584Asn), and p.(Ser1612Phe) could not be modeled.

### 2.4. Bile Acid Synthesis Is Not Affected by the CYP7A1 Variant p.(Pro398Ala) 

Cholestanol and 7α-hydroxy-cholesterol levels are significantly lower in carriers of p.(Pro398Ala) in CYP7A1, compared to non-carriers in the HC438 family (*p* = 0.015, *p* = 0.032, respectively), whereas bile acids levels are not affected (Table 3). This variant can therefore participate in the elevation of LDL-C levels. However, this cannot fully explain the phenotype, because family members II-5 and III-2 carrying this variant (II-5 and III-2) do not present hypercholesterolemia.

### 2.5. Contradictory Effects of LRP6 Variants on LRP6 Expression and LDL Binding and Uptake

**Figure 4:**
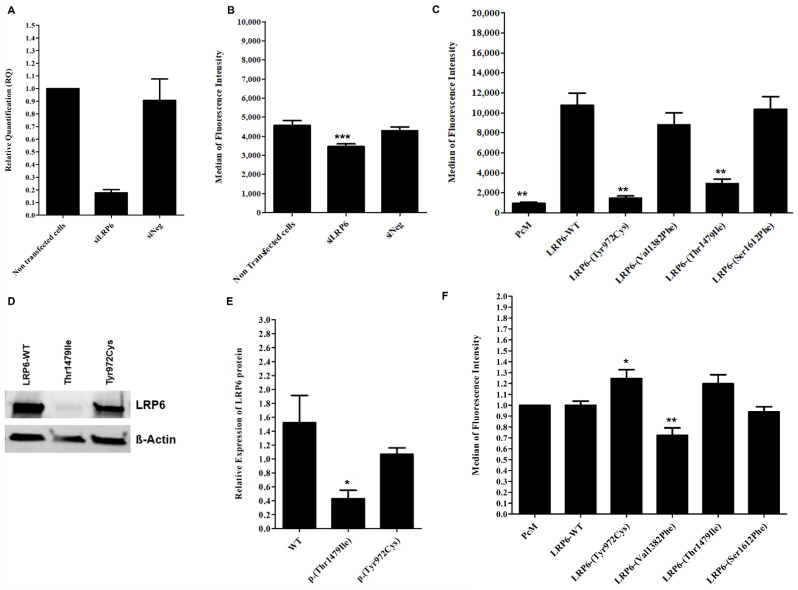
**Effect of inhibited, overexpressed or mutated *LRP6* in HEK293T and HuH7 cells**. (**A**) ***LRP6* mRNA expression in HuH7 after the silencing of *LRP6***. Reactions were run in triplicate for each cDNA. *POLR2A* was used as the reference housekeeping gene. The relative quantification of gene expression was performed using the ∆∆C_T_ method and non-transfected cells were used for calibration. (**B**) **LDL-Bodipy uptake in HuH7 after silencing of *LRP6***. Median fluorescence intensity of 50,000 events was acquired for each sample, but only the median fluorescence intensity of living cells is presented. Data represent three independent assays performed in triplicate. (**C**) **Expression of WT or mutated LRP6 at the cell surface of transfected HEK293T**. The median fluorescence intensity of 100,000 events was acquired for each sample, but only the median fluorescence intensity of living cells is presented. Data represent four independently performed assays. (**D**,**E**) **LRP6 expression in HEK293T cells after transfection with LRP6-WT or mutated plasmid (p.(Thr1479Ile) and p.(Tyr972Cys) variants)**. Proteins were extracted from transfected cells, separated by electrophoresis and then transferred onto PVDF membrane. The membrane was incubated with primary antibody (anti-LRP6), followed by incubation with secondary antibody before detection using the iBright^TM^ FL1500 imaging system. Protein was quantified by ImageJ software. Equal loading was confirmed using the ß-actin antibody. Data represent three independent assays. (**F**) **LDL uptake in HEK293T after transfection with an empty vector, LRP6-WT or mutated plasmid**. The median fluorescence intensity of 100,000 events was acquired for each sample, but only the median fluorescence intensity of living cells is presented. The fluorescence of each sample was normalized using the empty vector (PcM) as a reference. Data represent three independent assays, each performed in triplicate. In all experiments, the difference between conditions was determined by Bonferroni’s Multiple Comparison Test in one-way ANOVA and * *p* < 0.05, ** *p* < 0.01, *** *p* < 0.001 were considered as statistically significant. Results are shown as mean ± SD. Error bars represent ± SD.

HuH7 cells transfected with a siRNA targeting human *LRP6* (siLRP6) showed the expected reduced expression of the *LRP6* gene by 80% (Figure 4A). No significant differences in *LDLR*, *HMGCR*, *SREBP2* and *PCSK9* mRNA expression were observed (Figure S3), whereas labeled LDL uptake was significantly reduced by 23.14% and 20%, compared to non-transfected cells and to cells transfected with the negative control (siNeg), respectively (*p* < 0.05) (Figure 4B). 

As expected, cells transfected with the LRP6-WT plasmid present a significant increase in membrane expression of the LRP6 protein compared to cells transfected with the empty vector (PcM) (Figure 4C), but there is no significant difference in the binding and uptake of labeled LDL (Figure 4F and Figure S4). 

The two variants, p.(Tyr972Cys) and p.(Thr1479Ile), significantly decrease the surface expression of LRP6 compared to WT (*p* < 0.001) (Figure 4C), but only p.(Thr1479Ile) significantly decreases LRP6 protein expression in cells (Figure 4D,E). The two variants increase the binding and uptake of labeled LDL in HEK293T cells. However, a significant difference (*p* < 0.05) is observed only with the p.(Tyr972Cys) variant compared to LRP6-WT for binding and uptake measured simultaneously (Figure S4) and for uptake alone (Figure 4F). 

Unlike p.(Tyr972Cys) and p.(Thr1479Ile), p.(Val1382Phe) does not affect the membrane expression of LRP6 (Figure 4C), but it significantly reduces LDL uptake when compared to LRP6-WT (*p* < 0.05) (Figure 4F). Nevertheless, this variant has no significant effect when LDL binding and uptake were observed simultaneously (Figure S4).

The effect of LRP6-WT or the mutated plasmids on the membrane expression of the LDL receptor was also evaluated in transfected HEK293T cells and no significant changes were observed (Figure S5).

Theses confusing contradictory results suggest that each of these variants does not individually have a major effect on cellular LDL uptake and LDL-C circulating levels.

### 2.6. Heterozygotes Carriers of p.(Val1382Phe) Did Not Present Altered LDL Uptake or LRP6 Gene Expression

**Figure 5:**
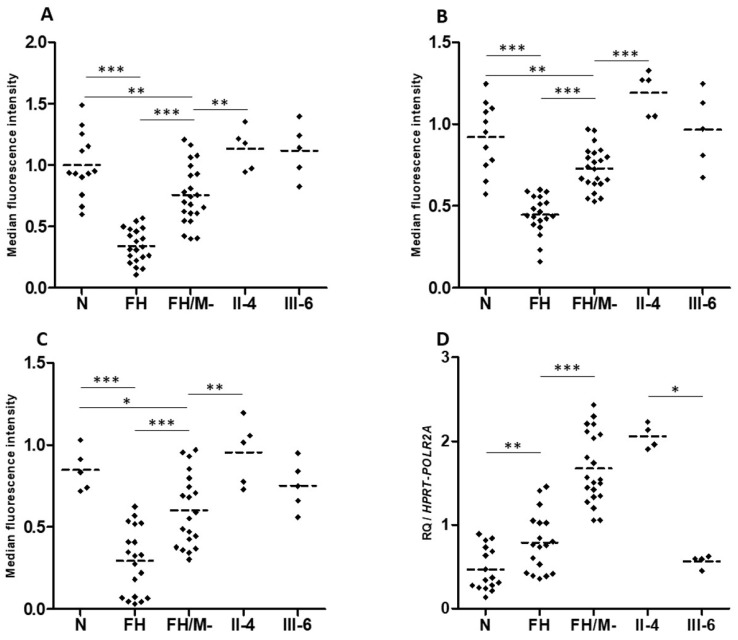
**LDL receptor expression, LDL binding and uptake, and LRP6 gene expression in patients EBV-transformed B-lymphocytes.** (**A**) LDL receptor, (**B**) LDL-Bodipy binding and (**C**) LDL-Bodipy uptake quantification in EBV-transformed B-lymphocytes from normocholesterolemic subjects (N), LDLR mutation carriers (FH), hypercholesterolemic patients without an identified mutation (FH/M-), and two *LRP6*-p.(Val1382Phe) carriers from the HC438 family: II-4 and III-6 (see Figure 1). The median fluorescence of living cells is presented. Data represent five independently performed assays. (**D**) LRP6 gene expression. Relative Quantification (RQ) of LRP6 in EBV-transformed B-lymphocytes. Reactions were run in triplicate for each cDNA. *HPRT* and *POL2RA* were used as reference genes. The relative quantification was performed using the ∆C_T_ method. (**A**–**D**). Bonferroni’s Multiple Comparison Test in one-way ANOVA: * *p* < 0.05, ** *p* < 0.01, *** *p* < 0.001.

The number of LDL receptors at the cell surface, LDL binding and LDL uptake are similar for EBV-transformed B-lymphocytes from two heterozygotes carriers of *LRP6*-p.(Val1382Phe), compared to cells from normocholesterolemic subjects. However, these parameters are significantly reduced in B-lymphocytes from FH (familial hypercholesterolemia) patients’ heterozygous for pathogenic variants in the *LDLR* gene (Figure 5A–C). This indicates that the LRP6-p.(Val1382Phe) variant alone does not affect the LDL cellular uptake. We hypothesize that the *CYP7A1*-p.(Pro398Ala) also carried by patient II.4 is not involved, since the *CYP7A1* gene is not expressed in the EBV-transformed B-lymphocytes.

Interestingly, *LRP6* gene expression is significantly higher in EBV-transformed B-lymphocytes from FH patients compared to cells from normocholesterolemic subjects (Figure 5D). Cells from affected patient II.4 present *LRP6* expression similar to that of cells from FH patients, whereas cells from unaffected carrier III-6 presented *LRP6* expression similar to that of cells from normocholesterolemic subjects (Figure 5D). This indicates that the higher *LRP6* gene expression is associated with the affected phenotype (FH and II.4 patients), but not with the *LRP6*-p.(Val1382Phe) variant carried by both family members II.4 and III.6.

### 2.7. Carriers of an LRP6 or LDLRAP1 Variant Present Lower LDL-C Levels than Non-Carriers Despite a Similar Polygenic Risk Score

In order to assess the contribution of polygenic hypercholesterolemia, we used a wPRS that includes risk alleles from the six frequent LDL-C-associated genes *CELSR2*, *APOB*, *ABCG5/8*, *LDLR* and *APOE* [26].

As expected, the mean LDL-C levels is significantly higher in the 152 ADH probands compared to the 13 non-ADH subjects (6.1 ± 1.5 vs. 3.4 ± 0.5 mmol/L, *p* < 0.0001) (Table 4). The mean LDL-C levels in the 4 carriers of a *CYP7A1* variants (5.5 ± 0.8 vs. 3.4 ± 0.5 mmol/L, *p* = 0.0053) and the 6 carriers of LRP6 variants (4.6 ± 1.3 vs. 3.4 ± 0.5 mmol/L, *p* = 0.0242) are significantly higher compared to those of the 13 non-ADH subjects (Table 4). The mean LDL-C levels in the 152 ADH probands were also significantly higher than those of the 6 carriers of LRP6 variants (4.6 ± 1.3 vs. 6.1 ± 1.5 mmol/L, *p* = 0.0088) (Table 4). These LDL-C differences were not attributable to a polygenic contribution, because the wPRS are statistically similar among the ADH probands and the carriers of variants in the three genes (Table 4). While our results are statistically significant, our variant group was limited to two or six subjects. Larger follow-up studies would confirm our results and add more statistical significance.

In the HC438 family, all the affected members present a wPRS in the top four deciles (VII, VIII, IX and X) of the WHII reference cohort, as well as six unaffected members (Figure 1). The wPRS does not influence the LDL-C levels in this family.

## 3. Discussion

We report here the first joint analysis of WES and WGS to identify ADH-causing genes in a French family. WES was performed for the three family members (I-2, II-1 and II-4) and WGS was performed for II-4 and the two additional family members II-6 and III-3. This permitted the analysis of five affected and unaffected members over three generations. WGS was used to complete WES with better genome coverage. The joint analysis permitted the filtering of variants according to segregation over the three generations with the retention of information and better accuracy compared to the separate analysis of the two sequencing results.

This approach allows us to identify ADH proband II-7, which has no detectable causal mutation in the four major ADH genes. Proband II-7 carries the three rare variants p.(Pro398Ala) in *CYP7A1*, p.(Val1382Phe) in *LRP6* and p.(Ser202His) in *LDLRAP1* in a polygenic background. The genotyping of the three variants with the wPRS calculation in the whole proband family allowed the identification of two affected members (I-2 and II-4) with the *CYP7A1*/*LRP6*/polygenic combination. None of the family members presenting a high wPRS and/or carrying only one of the variants in *LRP6*, *CYP7A1* or *LDLRAP1* was affected. To identify ADH carriers with oligogenic combinations, we also sequenced the 3 minor genes (*LRP6*, *CYP7A1* and *LDLRAP1*) in 160 unrelated ADH probands with no causal variants in the 4 major genes. We identified six additional rare variants. The two variants p.(Ala13Val) and p.(Asp347Asn) were found in *CYP7A1*, the three variants p.(Tyr972Cys), p.(Thr1479Ile) and p.(Ser1612Phe) were found in *LRP6* and the single variant p.(Ser202LeufsTer19) was found in *LDLRAP1*. No oligogenic combination of these variants was identified in the same proband. We then evaluated the effect of the p.(Pro398Ala) in *CYP7A1* on bile acid synthesis and observed no major effect, which would indicate a CYP7A1 deficiency. Similarly, the functional analysis of the four *LRP6* variants showed no major effect on LDL uptake. We concluded that each variant alone is insufficient to reveal the disease. Rather, a combination of variants is necessary.

The family proband II-7 had severe hypercholesterolemia, suggesting a homozygous form of ADH, and thus inherited defects from both parents. Whole genome and exome sequencing complemented by positional cloning analysis revealed the three rare variants *CD59* 3′UTR, *LDLRAP1* p.(Ser202His) and *GOLGA4* p.(Arg1494Ile) transmitted from the father and the four variants *CYP7A1* p.(Pro398Ala), *LRP6* p.(Val1382Phe), *SLC2A3* 3′UTR and *TSC2* intronic variant from the mother. We selected the variants in the three ADH minor genes *CYP7A1*, *LRP6* and *LDLRAP1* for further analysis in the whole family, but could not establish a good segregation with ADH for any of these genes. Indeed, each of the three variants is also carried by the unaffected family members II-8 and III-2 for p.(Pro398Ala) in *CYP7A1*, III-3 and III-6 for p.(Val1382Phe) in *LRP6* and I-1, II-8 and III-2 for p.(Ser202His) in *LDLRAP1*. Only the *CYP7A1* and *LRP6* variants are carried by the four affected family members, whereas the variants in *LDLRAP1* are present only in the two more severely affected children (II-1 and II-7). We concluded that the combined effects of p.(Pro398Ala) in *CYP7A1* and p.(Val1382Phe) in *LRP6* are needed to reveal the ADH phenotype. Furthermore, ADH becomes more severe with the addition of p.(Ser202His) in *LDLRAP1*.

While carrying the same genotype and being the more severely affected subjects, II-1 and II-7 have different LDL-C levels without treatment (7.95 vs. 10.73 mmol/L). The phenotypic variability in ADH is frequently reported as the consequence of metabolic, environmental and/or genetic factors [3]. The evaluation of the eating behavior and lifestyle of these two patients, as well as a search for modifying genetic factors, will help to understand this difference. However, the higher wPRS for subject II-7 (0.902 in decile X) compared to II-1 (0.831 in decile IX) may partially explain this phenotypic difference. The *CYP7A1* gene encodes cholesterol 7 alpha-hydroxylase, which is a 504 amino acid microsomal cytochrome P450 that catalyzes the rate-limiting reaction in the cholesterol catabolic pathway in the liver. This is the first step in the conversion of cholesterol to bile acids [34,35], and a deficiency of CYP7A1 would decrease bile acid production and the accumulation of cholesterol in the liver. This would lead to the retention of the Sterol Regulatory Element Binding Protein (SREBP) in the endoplasmic reticulum, the downregulation of LDL receptors, and consequent hypercholesterolemia. Several polymorphisms in *CYP7A1* were identified and associated with LDL-C levels [36,37]. Markers for cholesterol absorption, synthesis and degradation to bile acids as well as bile acids were measured in the serum of six members of the family. The results show that a marker of cholesterol absorption (cholestanol) and a marker of cholesterol degradation to bile acids (7α-hydroxy-cholesterol) are significantly lower in carriers of the p.(Pro398Ala) in *CYP7A1* compared to non-carriers (*p* = 0.015, *p* = 0.032, respectively), whereas bile acids are not affected. Thus, we concluded that p.(Pro398Ala) in *CYP7A1* can participate in the elevation of the LDL-C level; however, this variant cannot solely explain the ADH phenotype in the family. 

The human *LRP6* gene produces a 1613 amino acid protein that is a member of the LDL receptor family, which consists of transmembrane cell surface proteins involved in the receptor-mediated endocytosis of specific ligands [6,38]. The LRP6 receptor has a unique structure and is crucial for lipoprotein endocytosis. It also functions as an essential co-receptor for the Wnt/ß-catenin signaling pathway [19,20]. LRP6 has pleiotropic effects with an important role in cell differentiation, proliferation and migration during embryonic development. Pathogenic variants in *LRP6* are also associated with diverse human diseases [39]. The role of LRP6 in LDL metabolism was mainly studied by Mani et al., who identified the p.(Arg611Cys) missense variant in a family of Asian origin [40]. In addition to LRP6-(Arg611Cys), common variations within *LRP6* are associated with modest elevations in serum LDL in the general population. The common variant rs10845493 in *LRP6* is associated with elevated LDL levels [21] and rs2302685 (p.(Val1062Ile)) is associated with hypercholesterolemia [23]. The functional analysis of the four LRP6 variants identified in this study showed contradictory results.

We observed that the overexpression of *LRP6* in HEK293T cells had no significant effect on LDL uptake. This result differs from that of a previous study showing that overexpression of *LRP6* in CHO-K1 cells resulted in increased LDL internalization [38]. The discrepancy may be explained by the different cell types, HEK293T versus CHO-K1. Alternatively, our results show that the knockdown of *LRP6* in HuH7 cells reduce LDL uptake. This suggests that physiological levels of LRP6 are sufficient for LDL uptake in HEK293T cells and, consequently, the overexpression of LRP6 does not affect LDL uptake. 

The two *LRP6* variants p.(Tyr972Cys) and p.(Thr1479Ile) significantly decrease the membrane expression of LRP6 compared to WT. This is similar to the p.(Arg611Cys) variant in human lymphoblastoid cells [41] and CHO-K1 cells [38]. However, the decreases in the membrane expression of LRP6 does not affect the expression of the LDL receptor. This result is similar to that reported for the p.(Arg611Cys) variant in NIH3T3 and in human lymphoblastoid cells [41]. We tested the effects of the p.(Arg611Cys) variant in HEK293T cells and showed a slight but not significant decrease in membrane expression of LRP6, with no effect on the expression of the LDL receptor (data not shown). Moreover, p.(Tyr972Cys), which reduced the membrane expression of LRP6, significantly increased LDL uptake compared to LRP6-WT in HEK293T cells. These results were supported by the PyMOL modeling of *LRP6* p.(Tyr972Cys) that predicted conformational changes or altered interactions of LRP6 due to the loss of polar interaction with the neighboring Glu993 residue. The effect of p.(Tyr972Cys) on LDL uptake may be due to LRP6 competition with the LDL receptor in LDL uptake. The absence of LRP6 at the cell surface may increase LDL uptake by the LDL receptor. Nevertheless, this effect may not be major or sufficient to significantly reduce LDL levels in vivo.

The p.(Val1382Phe) variant is located in the transmembrane domain of LRP6 receptor and is probably unable to internalize after binding to LDL particles. However, the variant probably does not alter LDL binding or affect the membrane expression of LRP6. We conclude that this effect may not be major or sufficient to significantly increase LDL levels in vivo. 

Note that these newly identified variants in *LRP6* are not located in the same domain of the receptor and none is in the 2^nd^ EGF domain, which is specifically needed to induce the release of bound LDL at a low pH in the endosome [41] or the 2^nd^ ß-propeller domain where the previously described variants are associated with metabolic syndrome and CVD. 

The variant p. (Ser202His) in *LDLRAP1* was heterozygous in all carriers, and other heterozygous variants are known to increase LDL-C levels [15]. However, none of the family members carrying p. (Ser202His) alone presented elevated LDL-C levels, whereas the two unrelated probands with p.(Ser202His) and p.(Ser202LeufsTer19) presented elevated LDL-C levels.

Altogether, our results indicate that in the HC438 family, the combined effects of *CYP7A1*-p.(Pro398Ala) and *LRP6*-p.(Val1382Phe) are needed to reveal the ADH phenotype, which becomes more severe with the addition of the p.(Ser202His) in *LDLRAP1*. To our knowledge, no direct interactions between CYP7A1-related metabolism and the LRP6 or LDLRP1 pathways have been described. Functional studies showed that when the LDL particle binds to its receptor, LRP6 forms a complex with the LDL receptor, LDLRAP1 and clathrin to initiate endocytosis of the LDL receptor/LDL complex [19,38]. Thus, LRP6 and LDLRAP1 are essential for an efficient LDL endocytosis of the LDL. Furthermore, *LDLRAP1*-p.(Ser202His) probably worsens the effect of *LRP6*-p.(Val1382Phe), which leads to the more severe phenotype observed with the two affected family members II-1 and II-7 carrying the two variants. 

It would be interesting to functionally analyze both LRP6 and LDLRAP1. However, we have chosen to study the effect of each gene variant and performed the functional analyses of the four variants in *LRP6* alone. A future study will have to examine the functional analysis for both *LRP6*-p.(Val1382Phe) and *LDLRAP1*-p.(Ser202His). It will also be interesting to evaluate the effect of variants in the *CYP7A1*, *LRP6* or *LDLRAP1* genes on LDL-C levels and phenotypic variability among ADH patients with a pathogenic variant in a major ADH gene.

In summary, in the HC438 family, we identified three variants in three different ADH minor genes: p.(Pro398Ala) in *CYP7A1*, p.(Val1382Phe) in *LRP6* and p.(Ser202His) in *LDRAP1*. We excluded a major effect of each variant alone as well as the involvement of the wPRS. Thus, an oligogenic form of hypercholesterolemia is probably present in this family and high levels of LDL-C could be caused by the cumulative effect of *LRP6* and *CYP7A1* variants, which is aggravated by the *LDLRAP1* variant.

## 4. Materials and Methods

### 4.1. Probands and Family Recruitment

Probands and family members were all of non-Finnish European origin and recruited through the French Research Network on hypercholesterolemia that includes 14 different lipid clinics in France. Affected probands and family members meet the following inclusion criteria: total and LDL-C above the 90th percentile when compared with a sex- and age-matched French population (STANISLAS cohort [42]), normal levels of triglycerides and HDL-C and autosomal dominant transmission of hypercholesterolemia in the family. Exclusion criteria included any diseases leading to secondary hypercholesterolemia. Lipid levels before the initiation of the treatment were used when available. For all subjects, the four ADH-causing genes were studied as previously reported [30]. This allowed us to identify probands in whom mutations in *LDLR*, *APOB*, *PCSK9*, and *APOE* were excluded. Thus the study population consisted of 160 non-*LDLR*/non-*APOB*/non-*PCSK9*/non-*APOE* probands and 1 family (HC438).

DNA analyses in human subjects were performed after informed consent was obtained from all subjects in agreement with French bioethics laws. The research project received IRB approval (research project trial #05-07-06 approved by French Consultative Committee for the Protection of Person in Biomedical Research, Paris, Necker).

### 4.2. Whole Genome and Exome Sequencing and Data Analysis

Whole exome sequencing (WES) was performed at the Broad Institute of Harvard and MIT (Cambridge, MA, USA) for the three affected members I-2, II-1 and II-4 from the HC438 family (Figure 1), as previously described [43]. Whole genome sequencing (WGS) was performed at the Centre National de Recherche en Génomique Humaine (CNRGH, CEA, Evry, France) for the three family members (II-4, II-7 and III-4) using an Illumina HiSeq2500 platform. DNA was prepared using Illumina TruSeq DNA PCR-Free library preparation kits, according to the manufacturer’s instructions. An average sequencing depth of 30x was obtained for each sample. 

The VCF files from WES and WGS were analyzed conjointly using a dedicated in-house python pipeline. Variants were filtered according to (1) their quality (variant quality (Phred Qscore) > 20); (2) the genotype quality (>20) and depth (>5X); (3) the predicted consequence in terms of coding substitution and insertion/deletion (frameshift or in-frame), splice-site extending to 10 bases, UTR regions, and 1Kb upstream and downstream; (4) their frequency (minor allele frequency <0.003) based on the information available in the public databases (genome aggregation database (gnomAD) and 1000 genomes project version of August 2015); (5) their segregation with the disease in the 5 family members analyzed; and (6) their localization in a gene involved in at least 1 of the 7 pathways in which ADH genes are involved (Reactome (https://reactome.org (accessed on 6 November 2020)), which are cholesterol biosynthesis (R-HSA-191273), regulation of cholesterol biosynthesis by SREBP (R-HSA-1655829), clathrin-mediated endocytosis (R-HSA-8856828), vesicle-mediated transport (R-HSA-5653656), transport of small molecules (R-HSA-382551), digestion and absorption (R-HSA-8963743), bile acid and bile salt metabolism (R-HSA-194068)). 

Finally, variants in candidate genes were sequenced in the whole family to test their segregation with the disease under the hypothesis of an autosomal dominant inheritance.

### 4.3. Positional Cloning

In parallel with WES and WGS, a positional cloning approach using a genome-wide scan in 14 family members was performed with 1035 polymorphic microsatellite markers from deCODE Genetics, Iceland. Parametric linkage analyses were performed with accepted parameters for ADH, which were dominant transmission of the trait, penetrance of 0.6 for heterozygotes and a frequency of the disease allele of 0.01%. The power of the family for linkage was evaluated using the FastSlink v2.51 software [44]. We used Pedcheck [45] to detect Mendelian inheritance errors. SuperLink v1.5v [46] and SimWalk v2.91 [47] softwares were used to compute two-point and multipoint LOD scores. 

All these softwares were run using the easyLinkage Plus v5.00 package [48].

### 4.4. Sequencing and In Silico Analysis of the Variants

The coding exons of *LRP6*, *CYP7A1,* and *LDLRAP1* and their flanking exon–intron boundaries (100 pb surrounding each exon boundary) were sequenced by Sanger or by next-generation sequencing [49]. The coverage was >99% for the coding bases of the *LRP6* and *CYP7A1* genes and 90% for *LDLRAP1*, in the 160 unrelated ADH probands. The reference sequences were NM_002336.3 for *LRP6*, NM_000780.4 for *CYP7A1* and NM_015627.3 for *LDLRAP1* (GRCh37/hg19 in UCSC Genome Browser). 

Novel variants were mapped to known functional domains obtained using the UniProtKB database (UniProt Knowledgebase; www.uniprot.org (accessed on 6 November 2020)). The presence and frequency of these variants in a control group representative of the French population was verified using the French Exome Project database (FREX; www.france-genomique.org/bases-de-donnees/frex-the-french-exome-project-database/ (accessed on 12 November 2021)). Their frequency in the general population was taken from the Genome Aggregation database (gnomAD; http://gnomad.broadinstitute.org/ (accessed on 6 November 2020)). Their pathogenicity was evaluated using the Varsome tool (The Human Genomic Variant Search Engine; https://varsome.com (accessed on 12 November 2021)) [50], according to ACMG guidelines [33] and CADD score (Combined Annotation Dependent Depletion; https://cadd.gs.washington.edu/snv (accessed on 6 November 2020)). In addition, the in silico prediction tools PolyPhen-2 (Polymorphism Phenotyping version 2; https://genetics.bwh.harvard.edu/pph (accessed on 6 November 2020)), Provean (Protein Variation Effect Analyzer; https://provean.jcvi.org (accessed on 6 November 2020)), ClinVar (https://www.ncbi.nlm.nih.gov/clinvar/ (accessed on 6 November 2020)), and SpliceAI (https://spliceailookup.broadinstitute.org (accessed on 6 November 2020)) were used. 

### 4.5. Construction of LRP6 Structural Models

Structural models of the wild-type and mutant human LRP6-E3E4 (PDB ID 3S8Z) were generated using the PyMOL Molecular Graphics System, Version 2.0 Schrödinger, LLC (http://www.pymol.org (accessed on 6 November 2020)).

### 4.6. Weighted Polygenic Risk Score (wPRS) Calculation

For each individual, the wPRS was calculated using the weighted sum of the beta coefficient reported by the GLGC of the risk allele for the four selected SNPs plus the two *APOE* SNPs, as previously described [26,51]. wPRS were then compared to those of 3020 normocholesterolemic men and women of European ancestry from the U.K. Whitehall II (WHII) cohort study (SE Humphries and M Futema, personal communication). The probability of monogenic FH gradually increased for deciles under V, whereas scores in the top four deciles were associated with a high probability of polygenic hypercholesterolemia.

### 4.7. Sterol and Bile Acids Measurements

Markers of cholesterol absorption (campesterol, sitosterol, and cholestanol), cholesterol synthesis (lathosterol, lanosterol, and desmosterol), and cholesterol degradation to bile acids (7α-hydroxy-cholesterol, 27-hydroxy-cholesterol) as well as bile acids were measured in the patient’s serum with gas chromatography-mass spectrometry-selected ion monitoring, as previously described [52,53]. The values of non-cholesterol sterols and oxysterols were corrected for the cholesterol concentration (R_sterols). 

### 4.8. Site-Directed Mutagenesis

The coding sequence of *LRP6* (LRP6-WT) (NM_002336) is cloned into the 4.7 kb vector PCMV6XL4 (PcM), which encodes ampicillin resistance and is suitable for mammalian cell over-expression assays. The PcM and LRP6-WT plasmids were purchased from OriGene^®^ Technologies. Mutated plasmids were generated separately by site-directed mutagenesis using a Agilent^®^ Technologies QuickChange II XL Site-Directed Mutagenesis Kit, according to manufacturer’s instruction. Briefly, the mutant strand was synthesized by a thermal cycling reaction using a high-fidelity DNA polymerase and complementary mutagenic primers. This reaction was followed by a *DpnI* digestion of the parental methylated and hemimethylated DNA. The DNA vector containing the desired variant was then amplified into XL10-Gold^®^ Ultracompetent Cells by ThermoFisher^®^ Scientific and extracted using the NucleoBond^®^ Xtra Midi Plus kit by Machery-Nagel. The constructs were verified by Sanger sequencing with primers spanning the coding sequence of *LRP6*.

### 4.9. Cell Culture and Transfection

HuH7 cells were provided by Gael NICOLAS (INSERM U1149, CNRS ERL 8252, Centre de Recherche sur l’inflammation, Paris, France) and Hek293T cells were provided by ThermoFisher Scientific. HuH7 cells and HEK293T cells were authenticated by Eurofins Scientific. Cells were cultured in 1X Dulbecco’s modified Eagle medium (DMEM) supplemented with 10% FBS and 1% antibiotic-antimycotic (from Gibco^®^ by ThermoFisher^®^ Scientific, Waltham, MA, USA), at 37 °C under 5% CO_2_ in a humid atmosphere. Routine passage was every three days. For all experiments, HuH7 and HEK293T were used at passages 2 to 10. About 800,000 HEK293T cells were seeded in 6-well plates and maintained in complete DMEM medium (1X DMEM supplemented with 10% FBS) for 24 h before transfection. Cells were then co-transfected with the empty vector (PcM), LRP6-WT or mutated plasmids, and a plasmid containing cyan fluorescent protein (CFP or pSF-CMV-FrCFP from Oxford Genetics^®^, Oxford, U.K.) to assess the transfection rate by flow cytometry analysis. Cell transfections with a total of 3 µg of DNA per well were performed with Lipofectamine^®^ LTX and Plus^TM^ Reagent from Invitrogen^®^ by ThermoFisher^®^ Scientific in 1X DMEM medium supplemented with 10% FBS. At 24 h post-transfection, cells were starved for 24 h in serum-free media.

*LRP6* silencing was achieved using a Silencer^®^ Select siRNA targeting human *LRP6* (siLRP6) from Ambion. A siNeg from Eurogentec^®^ was used as a negative control. The siLRP6 sequence and siNeg sequence were not homologous to any other human gene sequence according to BLAST analysis. HuH7 cells were transfected with siLRP6 or siNeg using the reverse transfection method. Briefly, the siRNA molecule was diluted in 1X OptiMem Medium from Gibco^®^ by ThermoFisher^®^ Scientific in each well of a Biocoat Collagen I Cellware 6-well plate and combined with diluted Lipofectamine from Invitrogen^®^ by ThermoFisher^®^ Scientific to form complexes. About 400,000 HuH7 cells were suspended in 1X DMEM medium then added directly to the lipofectamine–siRNA complexes. Transfected HuH7 cells were maintained in the transfection medium until analysis.

Human EBV-transformed B-lymphocytes were cultured in suspension in RPMI-1640-glutamax medium supplemented with 10% FBS and 1% antibiotic-antimycotic from Gibco^®^ by ThermoFisher^®^ Scientific, at 37 °C under 5% CO_2_ in a humid atmosphere. The medium was partially renewed every three-to-seven days. About 2 × 10^6^ cells were seeded in 24-well plates and starved for 24 h in serum-free media before flow cytometry analysis or LDL-Bodipy treatment. About 5 × 10^6^ cells were seeded in 24-well plates. Each cell line was seeded in triplicate and starved for 24 h in serum-free media before RNA extraction.

### 4.10. LRP6 and LDL Receptor Cell Surface Expression

A human LRP6 APC-conjugated antibody was purchased from the R&D System and its specificity was confirmed in vitro by ELISA. The isotype control of LRP6, the PE mouse anti-human LDLR and its isotype control were purchased from BD Biosciences^®^. 

The cell surface expression of LRP6 and the LDL receptor in non-permeabilized and transfected HEK293T cells were analyzed by flow cytometry using antibodies that recognize the extracellular region of human LRP6 and human LDLR. The cell surface expression of the LDL receptor in human EBV-transformed B-lymphocytes was analyzed by flow cytometry using an antibody that recognized the extracellular region of human LDL receptor. 

At 48 h post-transfection with LRP6-WT or mutated plasmid, HEK293T cells were washed with 1X PBS, incubated with both LRP6 and LDL receptor antibodies for 30 min at 4 °C, then analyzed by flow cytometry using an LSRII from BD Bioscience^®^. 

At 24 h after incubation in serum-free DMEM medium, human EBV-transformed B-lymphocytes were washed, incubated with an LDL receptor antibody for 30 min at 4 °C and analyzed on a BD Accuri^TM^ C6 flow cytometer. 

Cell viability was assessed by LiveDead^®^ staining from Invitrogen^®^ by ThermoFisher^®^ Scientific. The median fluorescence intensity of 100,000 events was acquired for each sample, but only the median fluorescence intensity of living cells was analyzed. The assay was performed independently five times. 

### 4.11. LDL Uptake

Low-Density Lipoprotein from Human Plasma, Bodipy ^®^ FL complex (LDL-Bodipy) was purchased from ThermoFisher^®^ Scientific. 

The uptake of LDL in non-permeabilized and transfected HEK293T and HuH7 cells was analyzed by flow cytometry (LSRII from BD Bioscience^®^, Franklin Lakes, NJ, USA) using LDL-Bodipy. At 48 h post-transfection with LRP6-WT or mutated plasmid and 72 h post-transfection with siRNA, HEK293T and HuH7 cells were incubated in serum-free DMEM medium with 10 µg/mL LDL-Bodipy for 4 h at 37 °C. After four hours of incubation, the medium was removed, and the cells were washed twice with ice-cold 1X PBS. Labeled HEK293T and HuH7 cells were analyzed by flow cytometry. Cell viability was assessed by LiveDead^®^ in flow cytometry analysis. The median fluorescence intensity of 50,000 (siRNA transfection in HuH7) and 100,000 (plasmid transfection in HEK293T) events was acquired for each sample, but only the median fluorescence intensity of living cells was analyzed. Each assay was performed in triplicate and the triplicate assays were replicated independently three times.

The LDL binding and uptake of human EBV-transformed B-lymphocytes were analyzed by FACS using LDL-Bodipy. After incubation of the human EBV-transformed B-lymphocytes in a serum-free DMEM medium for 24 h, 10 µg/mL LDL-Bodipy was added and incubation was prolonged by 4 h at 4 °C for the binding experiment or at 37 °C for the uptake experiment. The cells were washed and analyzed on a BD Accuri™ C6 flow cytometer. Cell viability was assessed by LiveDead^®^ staining from Invitrogen^®^ by ThermoFisher^®^ Scientific. Median fluorescence intensity of 100,000 events was acquired for each sample, but only the median fluorescence intensity of living cells was analyzed. The assay was performed independently five times. 

### 4.12. Protein Extraction and Western Blot Assays

Transfected HEK293T cells were lysed in Pierce^®^ RIPA buffer from ThermoFisher^®^ Scientific supplemented with a Halt^TM^ Protease and Phosphatase Single-Use Inhibitor cocktail (100×) from ThermoFisher^®^ Scientific. Proteins were extracted by centrifugation for 15 min at 14,000× *g* at 4 °C. The total protein concentration was quantified on Tecan^®^ infinite 200 Pro using a Pierce^TM^ BCA Protein Assay kit from ThermoFisher^®^ Scientific.

Western blot assays were performed following the standard protocol. Equal quantities of protein extracts (2.5 μg) were loaded onto 4–20% Mini-Protean^®^ TGX^TM^ precast protein gels from BioRad^®^, separated by electrophoresis then transferred to Amersham hybond^TM^—P PVDF membrane from GE Healthcare^®^. The membrane was blocked with 10% non-fat dry milk in TBST 0.1% for one hour at room temperature then incubated overnight at 4 °C with a recombinant anti-LRP6 antibody (ab134146) from Abcam^®^. The membrane was washed three times with 0.1% TBST, followed by the addition of peroxidase-conjugated affiniPure Goat anti-Rabbit IgG secondary antibody from Jackson ImmunoResearch for one hour at room temperature. The membrane was then washed three times with 0.1% TBST, treated with the ECL detection kit (Clarity^TM^ Western ECL Substrate, from BioRAD^®^, Hercules, CA, USA) and detected using the iBright^TM^ FL1500 imaging system from ThermoFisher^®^ Scientific. Protein was quantified by ImageJ software (National Institutes of Health, Bethesda, MD, USA). An internal reference, ß-actin, was detected using a monoclonal anti-ß-actin-peroxidase antibody (AB3854) from Sigma-Aldrich. The results presented are representative of three independent experiments.

### 4.13. Total RNA Extraction, RT and Real-Time PCR Quantification

Total RNA was extracted from transfected HuH7 cells and human EBV-transformed B-lymphocytes using the RNeasy Mini Kit from Qiagen^®^ according to manufacturer’s instructions. RNA was eluted into RNase-free water, measured by Nanodrop spectrophotometer by Thermo Scientific^®^ to determine the concentration and quality, then stored at −80 °C until use. cDNA was produced using random primers and SuperScript^TM^ II Reverse Transcriptase from Invitrogen^®^ by ThermoFisher^®^ Scientific.

Messenger RNA expression levels of the *LRP6*, *LDLR*, *HMGCR*, *SREBP2*, and *PCSK9* genes were assessed using specific primers in HuH7 cells transfected with siRNA. mRNA expression levels of the *LRP6* gene were assessed in human EBV-transformed B-lymphocytes. Primers of target genes were mixed with Absolute Blue qPCR Mix, SYBR Green, ROX from ThermoFisher^®^ Scientific, and the cDNA solution was diluted to 2 ng/µL. Reactions were run in triplicate for each cDNA on an Applied^®^ Biosystems StepOnePlus^TM^ Real-Time PCR System. The data were analyzed using the StepOne software v2.3 and Microsoft Excel. Threshold cycle (C_T_) values were used to calculate the relative quantification (RQ) of gene expression using the comparative C_T_ (∆∆C_T_) method. Data were normalized using *POLR2A* as a reference gene. The observed differences were considered significant when the RQ is below 0.5 or above 2 [54]. The experiments were performed in triplicate.

### 4.14. Statistical Analysis

All variables are expressed as the mean ± standard deviation and represent the results of four independent experiments. Bonferroni’s Multiple Comparison Test in one-way ANOVA was used to assess the differences between the two groups. To ensure that variances were not significantly different between the groups, the Bartlett’s test was used. A probability value of *p* < 0.05 was considered significantly different. GraphPad Prism^®^ software was used for the statistical analysis and to generate the graphs.

## Figures and Tables

**Table 1:** **Variants in *CYP7A1*, *LDLRAP1* and *LRP6*.** The pathogenicity of the variants was evaluated using Varsome, PolyPhen2, Provean, ClinVar, CADD score and Splice AI.

Gene	c.notation p.notation	rs Number	Pathway	GTEx-TPM_Liver #	gnomAD (Total) *	gnomAD (ENF) *	FREX **	Varsome ***	PolyPhen2	Provean ^†^	ClinVar	CADD Score ^‡^	Splice AI
*CYP7A1* (NM_000780)	c.38C > T p.(Ala13Val)	rs147162838	Bile acid and bile salt metabolism	2.612	0.181% (512/282,726)	0.35% (451/129,062)	0.	LB	B	N (−0.189)	LB/VUS	7.125	No-consq (0)
	c.1039G > A p.(Asp347Asn)	rs8192875			0.274% (776/282,802)	0.019% (25/129,150)	…	LB	PD	D (−0.2990)	…	33	Donor gain (0.48)
	c.1192C > G p.(Pro398Ala)	rs142708991			0.336% (951/282,868)	0.43% (555/129,184)	0.0871%	LB	PD	D (−7.559)	LB	25.1	No-consq (0)
*LDLRAP1* (NM_015627)	c.603dupC p.(Ser202LeufsTer19)	rs781585299	Clathrin-mediated endocytosis	112.23	…	…	…	P	…	…	P	…	…
	c.604_605delTCinsCA p.(Ser202His)	rs386629678			…	…	…	LB	PD	N (−2.072)	LB	…	…
*LRP6* (NM_002336)	c.2915A > G p.(Tyr972Cys)	rs772441071	Vesicle-mediated transport	9.662	0.001193% (3/251,364)	0.002640% (3/113,656)	…	VUS	PD	D (−7.227)	…	26.9	Acceptor gain (0.04)
	c.4144G > T p.(Val1382Phe)	rs139480047			0.08379% (237/282,856)	0.1061% (137/129,164)	0.261%	B	B	N (−1.246)	LB	22.3	Donor gain (0.02)
	c.4436C > T p.(Thr1479Ile)	rs144175121			0.02263% (64/282,836)	0.04335% (56/129,168)	…	B	B	N (−1.906)	…	23.3	No-consq (0)
	c.4835C > T p.(Ser1612Phe)	…			0.0008097% (2/247,016)	0% (0/111,624)	…	VUS	PD	D (−2.879)	…	29.4	Donor gain (0.02)

# Gene expression in the liver, from the Genotype Tissue Expression database (GTEx). TPM: transcripts per million. * Allele frequency, from the Genome Aggregation Database (gnomAD): allele count/allele number in the general population and in the European non-Finnish (ENF). ** Allele frequency from the French Exome Project database. *** Varsome tool according to the ACMG guidelines [33]. ^†^ Provean: variant with a score of ≤−2.5 is considered “deleterious” and with a score of >−2.5 is considered “neutral”. ^‡^ CADD score ≥ 20 indicates that the variant is predicted to be among the top 1% of the most deleterious substitutions in the human genome, and a score of ≥30 indicates that the variant is predicted to be among the top 0.1% of the most deleterious substitutions in the human genome. N: neutral, LB: likely benign, B: benign, VUS: variant of unknown significance, PD: probably damaging, D: deleterious, P: pathogenic.

**Table 2:** Biological and clinical characteristics of the affected carriers of *CYP7A1, LDLRAP1* and *LRP6* variant.

Gene	Variant	Sex	Age *	TC **	LDL-C **	HDL-C **	TG **	wPRS	Decile	Clinic	Family History
*CYP7A1* (NM_000780)	c.38C > T p.(Ala13Val)	M	59	…	…	…	…	0.571	IV	…	…
	c.1039G > A p.(Asp347Asn)	F	71	7.92	6.24	…	0.98	0.25	I	…	…
	c.1192C > G p.(Pro398Ala)	M	71	7.52	5.69	…	…	…	…	…	…
*LDLRAP1* (NM_015627)	c.603dupC p.(Ser202LeufsTer19)	F	37	7.75	5.56	1.37	1.81	0.752	VII	…	…
	c.604_605delTCinsCA p.(Ser202His)	F	39	…	5.44	…	…	0.622	V	…	…
*LRP6* (NM_002336)	c.2915A > G p.(Tyr972Cys)	M ^†^	40	6.35	4.70	1.21	0.89	…	…	CAD	Yes Yes
		M ^‡^	32	7.47	5.30	1.34	1.83	0.371	II	No	
	c.4436C > T p.(Thr1479Ile)	F	48	8.15	5.57	2.12	1.01	0.581	IV	No	Yes
	c.4835C > T p.(Ser1612Phe)	M	51	8.04	5.89	1.71	0.94	0.542	III	CAD	Yes

* Age in years at lipid measurement. ** Lipid values (mmol/L) without lipid-lowering therapy. ^†^ Father. ^‡^ Son. TC: total cholesterol; LDL-C: low-density lipoprotein cholesterol; HDL-C: high-density lipoprotein cholesterol; TGs: triglycerides; wPRS: weighted polygenic risk score.

**Table 3:** Sterol and bile acid measurements in family HC438.

	II-1 *^,†^	II-4 **^,†^	II-5 ^†^	III-4	III-6	III-7	Carriers ***	Non-Carriers ***	Marker of	*p*-Value
Serum total cholesterol (mmol/L)	5.08	7.84	6.95	5.73	5.46	3.67	6.62 ± 1.41	4.95 ± 1.12		0.306
R_campesterol (μg/mg) **^‡^**	1.77	1.13	1.41	1.66	1.98	2.02	1.44 ± 0.32	0.32 ± 1.89	Cholesterol absorption	0.061
R_sitosterol (μg/mg) **^‡^**	1.65	0.87	0.97	0.94	1.26	1.30	1.16 ± 0.43	1.17 ± 0.20	Cholesterol absorption	0.495
R_cholestanol (μg/mg) **^‡^**	1.05	1.00	0.79	1.32	1.25	1.38	**0.95 ± 0.14**	**1.32 ± 0.07**	Cholesterol absorption	**0.015**
R_lathosterol (μg/mg) **^‡^**	0.68	1.46	1.39	2.02	1.22	2.01	1.18 ± 0.43	1.75 ± 0.46	Cholesterol synthesis	0.094
R_lanosterol (µg/mg) **^‡^**	0.10	0.14	0.12	0.17	0.11	0.20	0.12 ± 0.02	0.16 ± 0.05	Cholesterol synthesis	0.126
R_desmosterol (μg/mg) **^‡^**	0.65	0.78	0.77	0.91	0.58	0.77	0.74 ± 0.07	0.75 ± 0.17	Cholesterol synthesis	0.433
R_7αOH-cholesterol (ng/mg) **^‡^**	23	25	39	41	44	52	**29 ± 9**	**46 ± 6**	Degradation to bile acids	**0.032**
R_27OH-cholesterol (ng/mg) **^‡^**	113	108	95	107	117	128	105 ± 9	118 ± 10	Degradation to bile acids	0.104
Chenodeoxycholic acid (μmol/L)	3.05	2.76	1.34	2.11	0.01	3.49	2.38 ± 0.91	1.87 ± 1.75	Bile acids	0.342
Cholic acid (µmol/L)	1.76	1.92	0.44	1.74	0.01	1.28	1.38 ± 0.81	1.01 ± 0.89	Bile acids	0.314
Lithocholic acid (µmol/L)	0.06	0.16	0.21	0.20	0.31	0.11	0.14 ± 0.08	0.21 ± 0.10	Bile acids	0.224
Deoxycholic acid (µmol/L)	0.27	2.16	2.30	2.99	1.95	1.06	1.58 ± 1.13	2.00 ± 0.96	Bile acids	0.324

^†^ carriers of the variant p.(Pro398Ala) in *CYP7A1.* * under rosuvastatin 5 mg. ** under pravastatin 20 mg. **^‡^** values corrected for cholesterol concentration (R_sterols). *** Mean ± SD. Statistically significant differences between carriers and non-carriers are highlighted in bold (Bonferroni’s Multiple Comparison Test in one-way ANOVA).

**Table 4:** LDL-C levels and weighted Polygenic Risk Score (wPRS) comparison among the carriers of *CYP7A1*, *LRP6* and/or *LDLRAP1* variants.

	Non-ADH Subjects *	ADH Probands **	*CYP7A1* Variant Carriers ***	*LRP6* Variant Carriers ^#^	*LDLRAP1* Variant Carriers ^##^	*CYP7A1* and *LRP6* Variants Carriers ^###^	*CYP7A1*, *LRP6* and *LDLRAP1* Variants Carriers ^†^
N	13	152	4	6	3	2	2
Sex (% of women)	53.8	60.4	25	33.3	66.7	100	50
Age (years)	50 ± 15	48 ± 18	66 ± 6	39 ± 9	54 ± 28	81–71	6366
*p-value* vs. *non-ADH subjects*		*0.4897*	*0.0266*	*0.0520*	*0.3185*		
*p-value* vs. *ADH probands*			*0.0094*	*0.0453*	*0.4945*		
LDL-C (mmol/L)	3.4 ± 0.5	6.1 ± 1.5	5.5 ± 0.8	4.6 ± 1.3	4.7 ± 1.3	5.5–5.5	7.9–10.7
*p-value* vs. *non-ADH subjects*		*<0.0001*	*0.0053*	*0.0242*	*0.0693*		
*p-value* vs. *ADH probands*			*0.2364*	*0.0088*	*0.0542*		
wPRS	0.665 ± 0.165	0.700 ± 0.187	0.574 ± 0.326	0.655 ± 0.243	0.792 ± 0.193	0.731–0.831	0.831–0.902
*p-value* vs. *non-ADH subjects*		*0.2024*	*0.3428*	*0.500*	*0.2091*		
*p-value* vs. *ADH probands*			*0.2091*	*0.2548*	*0.2676*		

* Normocholesterolemic and non-carriers of a rare variant in an ADH-causing genes. ** Non-carriers of a rare variant in *LDLR*, *APOB*, *APOE*, *PCSK9*, *CYP7A1*, *LRP6*, and *LDLRAP1* genes. *** One p.(Ala13Val), one p.(Asp347Asn), and two p.(Pro398Ala) (Figure 1, Table 2). ^#^ Two p.(Tyr972Cys), two p.(Val1382Phe), one p.(Thr1479Ile), and one p.(Ser1612Phe) (Figure 1, Table 2). ^##^ One p.(Ser202LeufsTer19) and two p.(Ser202His) (Figure 1, Table 2). ^###^ Two p.(Pro398Ala) in *CYP7A1* and p.(Val1382Phe) in *LRP6* (Figure 1). ^†^ Two p.(Pro398Ala) in *CYP7A1*, p.(Val1382Phe) in *LRP6*, and p.(Ser202His) in *LDLRAP1* (Figure 1).

## Data Availability

All the data have been included in article.

## Supplementary Materials

The following are available online at https://www.mdpi.com/article/10.3390/metabo12030262/s1, Figure S1: Distribution of the probands in the 10 deciles of the weighted Polygenic Risk Score in the U.K. Whitehall II (WHII) cohort study; Figure S2: Conservation of the mutated amino acid in *LRP6*; Figure S3: Expression of genes implicated in cholesterol metabolism in HuH7 cells transfected with siLRP6; Figure S4: LDL binding and uptake in HEK293T after transfection with an empty vector, LRP6-WT or mutated plasmid (cells harvested without trypsin); Figure S5: Effects of transfection with WT or mutated LRP6 on membrane expression of LDL receptors; Table S1: Variants identified in the HC438 family by WES and WGS; Table S2: Linkage analysis of the variants identified in the HC438 family by WES and WGS.
