# Supplementary material for: Whole Exome/Genome Sequencing Joint Analysis of a Family with Oligogenic Familial Hypercholesterolemia
Source: Metabolites. 2022 Mar 18;12(3):262. doi: 10.3390/metabo12030262 (PMC8955453; doi:10.3390/metabo12030262)
Supplement: Supplementary file 1 [file metabolites-12-00262-s001.zip › metabolites-1586567-supplementary.pdf]

1. Supplementary Figures

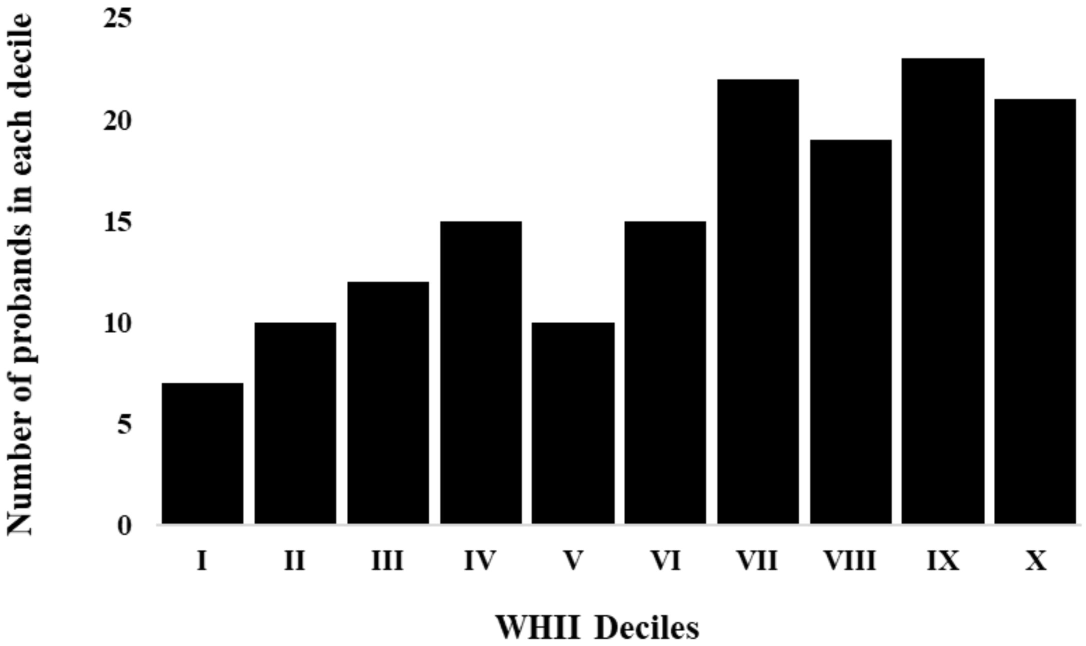

Figure S1. Distribution of probands in the 10 deciles of the weighted Polygenic Risk Score (wPRS) in the UK Whitehall II (WHII) cohort study.

(**A**)

	GTT	ATT	GGC	GTA	ATT	GTC	ACC	ATT	TTT	GTG	TCT
Human	V	I	G	V	I	V	T	I	F	V	S
Rhesus	V	I	G	V	I	V	T	I	F	V	S
Mouse	V	I	G	V	I	V	T	I	F	V	S
Dog	V	I	G	V	I	V	T	I	F	V	S
Elephant	V	I	G	V	I	V	T	I	F	V	S
Chicken	V	I	G	V	I	L	T	I	F	V	S
Zebrafish	V	I	G	V	I	L	T	I	F	V	S

(**B**)

	GTC	CGG	GCC	ATT	GAC	TAT	GAC	CCA	CTG	GAC	AAG
Human	V	R	A	I	D	Y	D	P	L	D	K
Rhesus	V	R	A	I	D	Y	D	P	L	D	K
Mouse	V	R	A	I	D	Y	D	P	L	D	K
Dog	V	R	A	I	D	Y	D	P	L	D	K
Elephant	V	R	A	I	D	Y	D	P	L	D	K
Chicken	V	R	A	I	D	Y	D	P	L	D	K
Zebrafish	V	R	A	I	D	F	D	P	L	D	K

(**C**)

	AGC	ACC	AAA	GGC	ACT	TAC	TTC	CCT	GCA
Human	S	T	K	G	T	Y	F	P	A
Rhesus	S	T	K	G	T	Y	F	P	A
Mouse	S	T	K	G	T	Y	F	P	A
Dog	S	T	K	G	T	Y	F	P	A
Elephant	S	T	K	G	T	Y	F	P	A
Chicken	S	T	K	G	T	Y	F	P	P
Zebrafish	S	T	K	G	A	F	Y	P	Q

(**D**)

	TCT	CCC	TGT	ACA	GAC	TCC	TCC	TGA
Human	S	P	C	T	D	S	S	*
Rhesus	S	P	C	T	D	S	S	*
Mouse	S	P	C	T	D	S	S	*
Dog	S	P	C	T	D	S	S	*
Elephant	S	P	C	T	D	S	S	*
Chicken	S	P	C	T	D	S	S	*
Zebrafish	S	P	C	T	D	S	S	*

Figure S2. Conservation of the mutated amino acid in *LRP6*. The conservation of the mutated amino acid between species from human to zebrafish is represented for each variant. The position of the variant is indicated in red. (**A**) p.(Val1382Phe) variant. (**B**) p.(Tyr972Cys) variant. (**C**) p.(Thr1479Ile) variant. (**D**) p.(Ser1612Phe) variant.

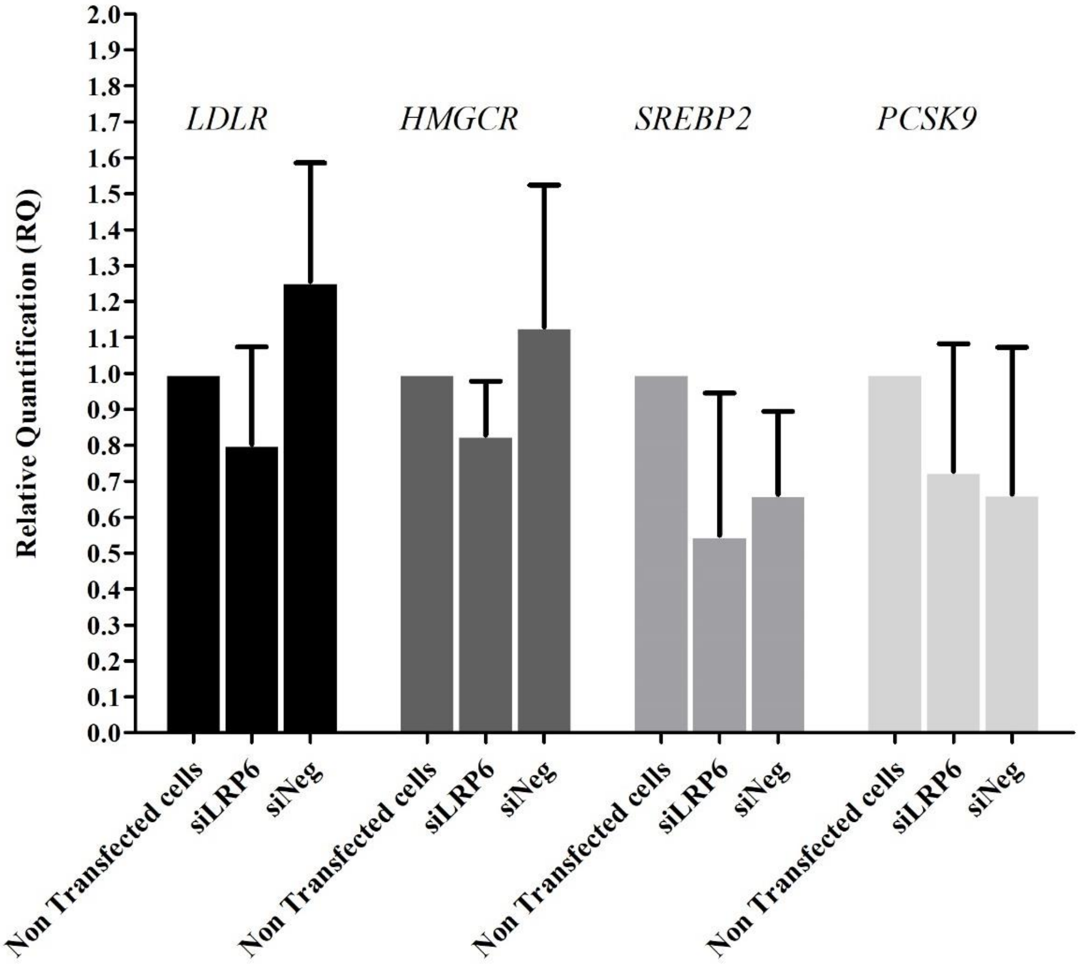

Figure S3. Expression of genes implicated in cholesterol metabolism in HuH7 cells transfected with siLRP6. Mean Relative Quantification (RQ) for *LDLR*, *HMGCR*, *SREBP2* and *PCSK9* in HuH7 cells transfected with siLRP6, siNeg and in non-transfected HuH7 cells. Reactions were run in triplicate for each cDNA. *POLR2A* was used as the reference gene. The relative quantification of gene expression was performed using the $\Delta\Delta C_T$ method and the non-transfected cells were used as calibrator. Results are shown as mean ± standard deviation (SD).

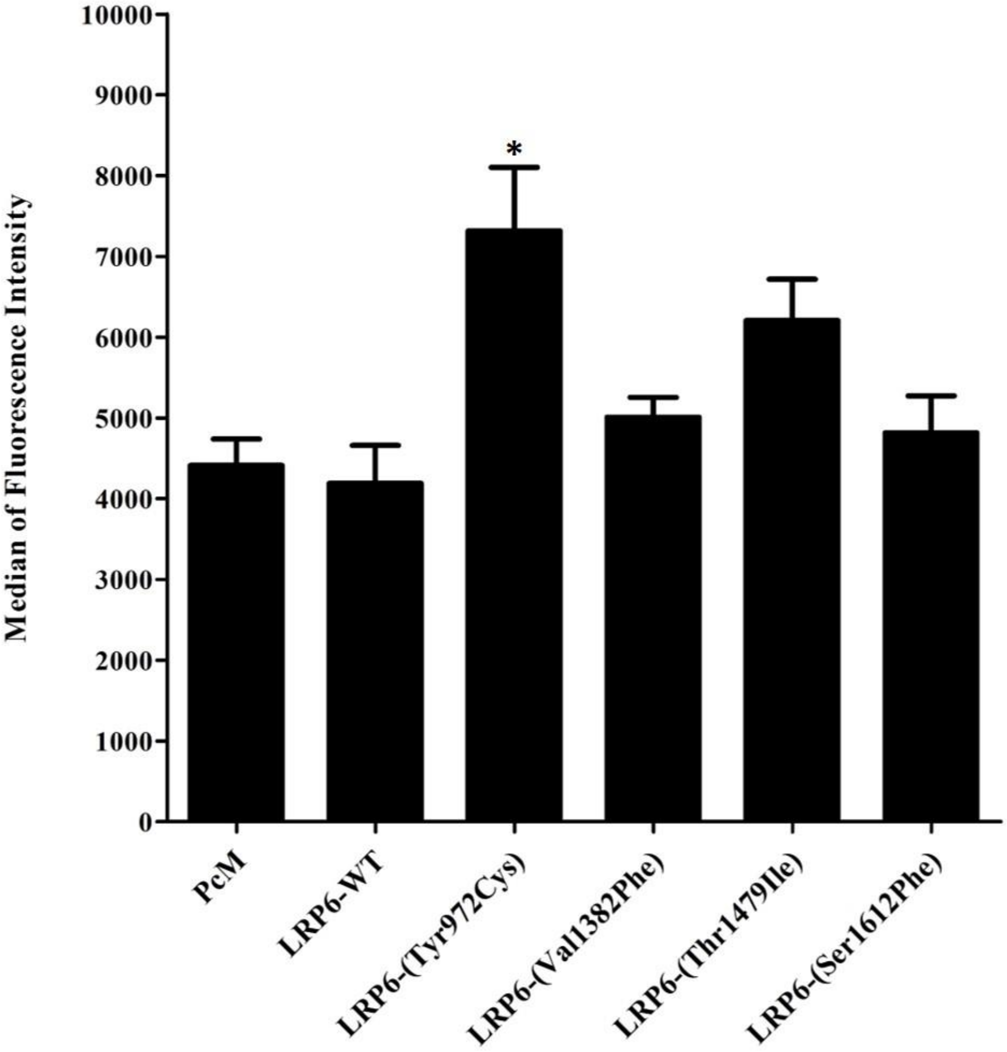

Figure S4. LDL binding and uptake in HEK293T after transfection with empty vector, LRP6-WT or mutated plasmid (cells harvested without trypsin). Median fluorescence intensity of 100000 events was acquired for each sample but only median fluorescence intensity of living cells is presented. Data represent four independent assays. In all experiments, the difference between two conditions was determined by non-parametric Mann-Whitney U test and p-values < 0.05 were considered as statistically significant. Error bars represent ± SD.*: $p < 0.05$

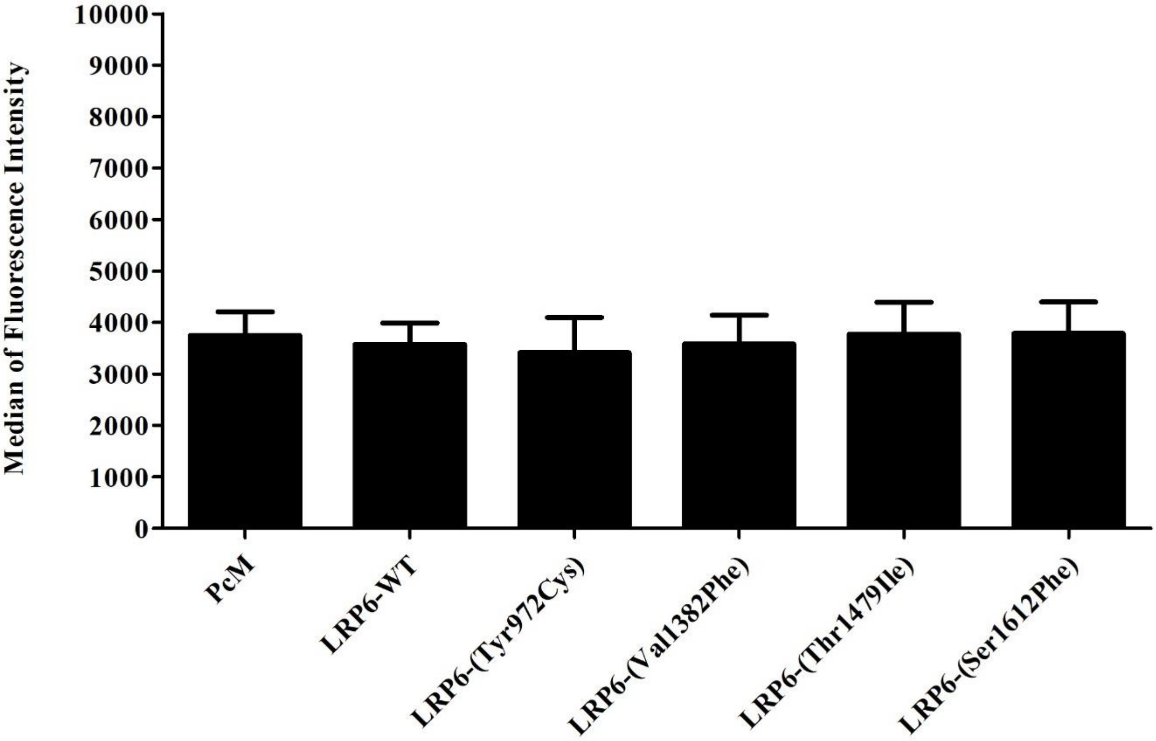

Figure S5. Effects of transfection with WT or mutated LRP6 on membrane expression of LDL receptor. Membrane expression of LDL receptor in HEK293T cells after transfection with empty vector, LRP6-WT or mutated plasmid. Cells were transfected with the corresponding plasmid for 48 hours, labeled with extracellular PE mouse anti-human LDLR antibody for 30 minutes at 4°C then analyzed by FACS. Median fluorescence intensity of 100000 events was acquired for each sample but we represent only media fluorescence intensity of living cells. Data represent four independently performed assays. The difference between cells transfected with empty vector, LRP6-WT or mutated plasmid was determined by non-parametric Mann-Whitney U test and p-values < 0.05 were considered as statistically significant. Error bars represent ± SD.

2. Supplementary Tables.

Table S1. Variants identified in HC438 family by Whole Exome/Genome Sequencing. The pathogenicity of the variants was evaluated using Varsome, PolyPhen2, Provean, ClinVar, CADD score, and Splice AI.

Gene	c.notation p.notation	rs number	Pathway	GTEx-TPM_Liver #	gnomAD (total)*	gnomAD (ENF)*	FREX**	Varsome***	PolyPhen 2	Provean †	ClinVar	CADD Score ‡	Splice AI
**Nonsynonymous Variants transmitted from I-2 to II-1, II-4 and II-7**													
CYP7A1 (NM_000780)	c.1192C>G p.(Pro398Ala)	rs142708991	Bile acid and bile salt metabolism	2.612	0.336% (951/282868)	0.43% (555/129184)	0.0871 %	LB	PD	D (-7.559)	LB	25.1	No-consq (0)
KIFC2 (NM_145754)	c.1399C>T p.(Pro467Ser)	…	Vesicle-mediated transport	11.75	…	…	…	VUS	B	N (-2.482)	…	19.14	No-consq (0)
LRP6 (NM_002336)	c.4144G>T p.(Val1382Phe)	rs139480047	Vesicle-mediated transport	9.662	0.08379% (237/282856)	0.1061% (137/129164)	0.261%	B	B	N (-1.246)	LB	22.3	Donor gain (0.02)
**Intronic Variant transmitted from I-2 to II-1, II-4 and II-7**													
SLC39A4 (NM_017767.3)	c.1074+10C>T	rs782654111	Transport of small molecules	2.835	0.0072% (17/237238)	0.000093% (1/107736)	…	LB	…	…	B	5.199	Donor Loss (0.01)
**Nonsynonymous Variants transmitted from I-1 to II-1 and II-7**													
LDLRAP1 (NM_015627)	c.604_605delTCinsCA p.(Ser202His)	rs386629678	Clathrin-mediated endocytosis	112.23	…	…	…	LB	PD	N (-2.072)	LB	…	…
GOLGA4 (NM_2078)	c.4481G>T p.(Arg1516Ile)	rs201034947	Vesicle-mediated transport	17.45	…	…	…	VUS	PD	D (-2.826)	…	14.84	No-consq (0)
AP2A1 (NM_130787)	c.1927C>T p.(Gly643Ser)	…	Vesicle-mediated transport	26.45	…	…	…	VUS	B	N (0.111)	…	19.11	Donor Loss (0.01)
**Nonsynonymous Variant transmitted from I-2 to II-1 and II-7**													

MOGAT2 (NM_025098)	c.14C>T p.(Ala5Val)	rs146774065	Digestion and absorption	57.92	0.0059% (9/152220)	0% (0/68036)	…	LB	PD	D (-2.916)	…	19.18	No-consq (0)
**Intronic Variants transmitted from I-2 to II-1 and II-7**													
PEX19 (NM_2857.4)	c.181-5C>T	rs199818690	Transport of small molecules	30.73	0.100% (154/152170)	0.180% (122/68030)	0.2	VUS	…	…	LB/VUS	1.094	Acceptor Loss (0.02)
TSC2 (NM_0548.5)	c.2838-4A>G	rs45517272	Vesicle-mediated transport	17.91	0.085% (240/282284)	0.12% (156/128748)	…	B	…	…	B	12.26	Acceptor Loss (0.08)
**5′UTR Variants transmitted from I-1 or I-2 to II-1, II-4 and II-7 ++**													
AP3S1 (NM_1284)	c.-114T>G	rs62371472	Vesicle-mediated transport	33.86	…	…	…	VUS/B	…	…	…	15.23	No-consq (0)
AP2A2 (NM_012305)	c.-49G>C	…	Vesicle-mediated transport	20.46	…	…	…	VUS	…	…	…	5.6	No-consq (0)
**3′UTR Variants transmitted from I-1 or I-2 to II-1, II-4 and II-7 ++**													
SMAP2 (NM_022733)	c.*1073C>T	rs541351955	Vesicle-mediated transport	29.56	0.0032% (1/31392)	0.000 (0/15424)	…	VUS	…	…	…	9.1	…
SEC22B (NM_004892)	c.*425T>G	…	Vesicle-mediated transport	9.244	0.00068% (1/146068)	0.000 (0/65958)	…	VUS	…	…	…	5.745	No-consq (0)
ACTR3 (NM_005721)	c.*3247C>T	rs1322808539	Vesicle-mediated transport	12.07	…	…	…	VUS	…	…	…	3.857	Acceptor Gain (0.01)
SEC22C (NM_032970)	c.*2598A>G	…	Vesicle-mediated transport	3.841	…	…	…	VUS	…	…	…	3.241	Donor Gain (0.03)
KIF13B (NM_015254)	c.*2477A>C	rs879249361	Vesicle-mediated transport	5.813	…	…	…	VUS	…	…	…	3.241	No-consq (0)
CD59 (NM_001127223)	c.*3923A>G	…	Vesicle-mediated transport	65.13	…	…	…	VUS	…	…	…	3.169	No-consq (0)

SLC2A3 (NM_006931)	c.*1345dup	rs1335777343	Transport of small molecules	7.774	0.002% (3/152086)	0.003% (2/68026)	…	VUS	…	…	…	…	…
RAB27A (NM_004580)	c.*2253_*2256delTTTG	…	Vesicle-mediated transport	12.7	…	…	…	VUS	…	…	…	…	…
AQP9 (NM_020980)	c.*1766G>A	rs551728298	Bile acid and bile salt metabolism	110	0.0032% (1/31376)	0.00 (0/15420	…	VUS	…	…	…	1.264	Acceptor Loss (0.01)
MED1 (NM_004774)	c.*923T>G	rs988981826	Regulation of cholesterol biosynthesis by SREBP	3.617	…	…	…	VUS/B	…	…	…	15.96	No-consq (0)

Gene expression in the liver, from the Genotype Tissue Expression database (GTEx). TPM: transcripts per million
* Allele frequency, from the Genome Aggregation database (gnomAD): allele count/allele number in the general population and in the European non-Finnish
** Allele frequency from the French Exome Project database.
*** Varsome tool according to the ACMG guidelines [51]
++ 3'UTR and 5'UTR variants were uncovered by the WES in subjects I-2 and II-1.
† Provean: Variant with a score ≤-2.5 is considered "deleterious" and with a score > -2.5 is considered "neutral".
‡ CADD score ≥ 20 indicates that the variant is predicted to be among the top 1% of the most deleterious substitutions in the human genome, and a score ≥ 30 indicates that the variant is predicted to be among the top 0.1% of the most deleterious substitutions in the human genome.
N: neutral, LB: likely benign, B: benign, VUS: variant of unknown significance, PD: probably damaging, D: deleterious

Table S2. Linkage analysis of the variants identified in HC438 family by Whole Exome/Genome Sequencing.

Chromosome	Gene	c.notation p.notation	rs number	Multipoint LOD SCORE	Results
Hypothesis 1: A paternal trait inherited by the three affected children. mean ELOD=0.72, max ELOD=1.84					
1	*SMAP2* (NM_022733)	c.*1073C>T	rs541351955	-1.417	Probably excluded
1	*SEC22B* (NM_4892)	c.*425T>G	…	-3.704	Excluded
2	*ACTR3* (NM_5721)	c.*3247C>T	rs1322808539	0.067	…
3	*SEC22C*	c.*2598A>G	…	0.418	…

	(NM_032970)				
5	*AP3S1* (NM_1284)	c.-114T>G	rs62371472	-2.688	Excluded
8	*KIF13B* (NM_015254)	c.*2477A>C	rs879249361	-0.951	…
11	*AP2A2* (NM_012305)	c.-49G>C	…	0.519	…
11	*CD59* (NM_1127223)	c.*3923A>G	…	**0.914**	**Probably linked**
12	*SLC2A3* (NM_6931)	c.*1345dup	rs1335777343	-3.154	Excluded
15	*AQP9* (NM_020980)	c.*1766G>A	rs551728298	0.966	…
15	*RAB27A* (NM_4580)	c.*2253_*2256delCAAA	…	0.862	…
17	*MED1* (NM_4774)	c.*923T>G	rs988981826	0.467	…
Hypothesis 2: A paternal trait inherited by the two more severely affected children. mean ELOD=0.51, max ELOD=1.61					
1	*LDLRAP1* (NM_015627)	c.604_605delTCinsCA p.(Ser202His)	rs386629678	**0.813**	**Probably linked**
3	*GOLGA4* (NM_2078)	c.4481G>T p.(Arg1494Ile)	rs201034947	**1.198**	**Probably linked**
19	*AP2A1* (NM_130787)	c.1927C>T p.(Gly643Ser)	…	0.417	…
Hypothesis 3: A maternal trait inherited by the three affected children. mean ELOD=0.77, max ELOD=2.00					
1	*SMAP2* (NM_022733)	c.*1073C>T	rs541351955	-1.278	Probably excluded
1	*SEC22B* (NM_4892)	c.*425T>G	…	0.150	…
2	*ACTR3* (NM_5721)	c.*3247C>T	rs1322808539	-0.072	…
3	*SEC22C* (NM_032970)	c.*2598A>G	…	-3.171	Excluded
5	*AP3S1* (NM_1284)	c.-114T>G	rs62371472	-3.259	Excluded
8	*CYP7A1*	c.1192C>G	rs142708991	**1.200**	**Probably linked**

	(NM_000780)	p.(Pro398Ala)			
8	*KIFC2* (NM_145754)	c.1399C>T p.(Pro467Ser)	…	0.200	…
8	*SLC39A4* (NM_017767.3)	c.1074+10C>T	rs782654111	0.201	…
8	*KIF13B* (NM_015254)	c.*2477A>C	rs879249361	-0.770	…
11	*AP2A2* (NM_012305)	c.-49G>C	…	-0.376	…
11	*CD59* (NM_1127223)	c.*3923A>G	…	-4.332	Excluded
12	*LRP6* (NM_002336)	c.4144G>T p.(Val1382Phe)	rs139480047	**1.369**	**Probably linked**
12	*SLC2A3* (NM_6931)	c.*1345dup	rs1335777343	**1.042**	**Probably linked**
15	*AQP9* (NM_020980)	c.*1766G>A	rs551728298	-3.820	Excluded
15	*RAB27A* (NM_4580)	c.*2253_*2256delCAAA	…	-2.796	Excluded
17	*MED1* (NM_4774)	c.*923T>G	rs988981826	0.467	…
Hypothesis 4: A maternal trait inherited by the two more severely affected children. mean ELOD=0.54, max ELOD=1.61					
1	*PEX19* (NM_2857.4)	c.181-5C>T	rs199818690	0.419	…
11	*MOGAT2* (NM_025098)	c.14C>T p.(Ala5Val)	rs146774065	-0.492	…
16	*TSC2* (NM_0548.5)	c.2838-4A>G	rs45517272	**0.673**	**Probably linked**

ELOD = expected LOD SCORE.
